# Macrophage inhibitory cytokine-1 aggravates diet-induced gallstone formation via increased ABCG5/ABCG8 expression

**DOI:** 10.1371/journal.pone.0287146

**Published:** 2023-06-13

**Authors:** Min Hee Kim, Eun-Ji Lee, Su-Jeong Kim, Yun-Jae Jung, Woo-Jae Park, Inkeun Park

**Affiliations:** 1 Department of Biochemistry, College of Medicine, Ewha Womans University, Seoul, Republic of Korea; 2 Department of Biochemistry, Chung-Ang University College of Medicine, Seoul, Republic of Korea; 3 Department of Microbiology, Lee Gil Ya Cancer and Diabetes Institute, College of Medicine, Gachon University, Incheon, Republic of Korea; 4 Department of Health Science and Technology, Gachon Advanced Institute for Health Science & Technology, Gachon University, Incheon, Republic of Korea; 5 Department of Oncology, Asan Medical Center, University of Ulsan College of Medicine, Seoul, Republic of Korea; University of Alberta, CANADA

## Abstract

Macrophage inhibitory cytokine 1 (MIC-1), which is overproduced in various human cancers and associated with cachexia, acts on the hypothalamus to suppress appetite and reduce body weight. We investigated the mechanisms through which MIC-1 affects bile acid metabolism and gallstone formation, which are poorly understood. Over 6 weeks, male C57BL/6 mice fed either standard chow or a lithogenic diet were intraperitoneally injected with phosphate-buffered saline (PBS) or MIC-1 (200 μg/kg/week). Among lithogenic diet–fed mice, MIC-1 treatment resulted in increased gallstone formation compared with PBS treatment. Compared with PBS treatment, MIC-1 treatment decreased hepatic cholesterol and bile acid levels and reduced expression of HMG-CoA reductase (HMGCR), the master cholesterol metabolism regulator sterol regulatory element-binding protein 2, cholesterol 7α-hydroxylase (CYP7A1), mitochondrial sterol 27-hydroxylase, and oxysterol 7α-hydroxylase. Compared with PBS treatment, MIC-1 treatment had no effect on small heterodimer partner, farnesoid X receptor, or pregnane X receptor expression, and extracellular signal–related kinase and c-Jun N-terminal kinase phosphorylation decreased, suggesting that these factors do not contribute to the MIC-1–induced reduction in CYP7A1 expression. Compared with PBS treatment, MIC-1 treatment increased AMP-activated protein kinase (AMPK) phosphorylation. Treatment with the AMPK activator 5-aminoimidazole-4-carboxamide ribonucleoside (AICAR) reduced CYP7A1 and HMGCR expression, whereas the AMPK inhibitor Compound C reversed MIC-1-induced reductions in CYP7A1 and HMGCR expression. Furthermore, in MIC-1-treated mice, total biliary cholesterol levels increased together with increased ATP-binding cassette subfamily G (ABCG)5 and ABCG8 expression. Compared with PBS treatment, MIC-1 treatment did not affect expression of liver X receptors α and β, liver receptor homolog 1, hepatocyte nuclear factor 4α, or NR1I3 (also known as constitutive androstane receptor), which are upstream of ABCG5/8; however, MIC-1 treatment increased ABCG5/8 expression and promoter activities. Our study indicates that MIC-1 influences gallstone formation by increasing AMPK phosphorylation, reducing CYP7A1 and HMGCR expression, and increasing ABCG5 and ABCG8 expression.

## Introduction

Gallstones can be classified into two types that form through completely different pathogenic mechanisms: cholesterol stones or pigment stones (black and brown) [1]. Cholesterol stones are the most commonly occurring type of gallstone and are often asymptomatic [2]. Gallstones are generated in response to interactions between lithogenic polymorphisms in several genes and various environmental factors, but the genes responsible for gallstone production in humans have not been well studied [3]. Cholesterol gallstones form when cholesterol precipitates under conditions of cholesterol supersaturation due to the hypersecretion of bile cholesterol, low bile acid secretion, and low phospholipid secretion [2].

Two ATP-binding cassette (ABC) transporters, ABCG5 and ABCG8, are expressed by hepatocytes. Positive regulators, including liver X receptor (LXR), liver receptor homolog 1 (LRH1), hepatocyte nuclear factor 4 alpha (HNF4α), and NR1I3 (also known as the constitutive androstane receptor) [4–8], increase ABCG5 and ABCG8 expression, which facilitates biliary cholesterol secretion and increases hepatic cholesterol synthesis [4, 5]. Therefore, ABCG5 and ABCG8 are thought to play crucial roles in gallstone formation by regulating cholesterol secretion [9].

The rate-limiting enzyme of cholesterol synthesis is 3-hydroxy-3-methylglutaryl-coenzyme A (HMG-CoA) reductase (HMGCR), which converts HMG-CoA to mevalonate [10] and is regulated by sterol regulatory element-binding protein (SREBP) [11]. SREBP is a membrane-bound transcription factor that controls cholesterol and fatty acid biosynthesis and has three isoforms: SREBP1a, SREBP1c, and SREBP2 [10]. SREBP2 is primarily involved in cholesterol synthesis, SREBP1c is predominantly involved in fatty acid synthesis in the liver [12], and SREBP1a can activate both pathways [11]. SREBP2 induces cholesterol biosynthesis by increasing the expression levels of HMGCR, HMG-CoA synthase, and mevalonate kinase and promotes cholesterol uptake by increasing the expression of low-density lipoprotein receptor. SREBP1c induces fatty acid synthesis by increasing the expression of fatty acid synthase (FAS) and acetyl-CoA carboxylase (ACC) [12]. AMP-activated protein kinase (AMPK) is a serine/threonine protein kinase that acts as a cellular energy sensor [13] and regulates energy homeostasis [14]. AMPK activation in the liver inhibits ACC, FAS, and HMGCR activities, inhibiting fatty acid and cholesterol synthesis [13, 14].

Cholesterol 7α-hydroxylase (CYP7A1) is a rate-limiting enzyme in the bile acid biosynthesis pathway in the liver [15], which is also regulated by enzymes such as mitochondrial sterol 27-hydroxylase (CYP27A1), oxysterol 7α-hydroxylase (CYP7B1), and sterol 12α-hydroxylase (CYP8B1) [16]. Many nuclear receptors, including farnesoid X receptor (FXR), pregnane X receptor (PXR), and small heterodimer partner (SHP), act as transcription factors that regulate genes involved in bile acid synthesis [16], such as *CYP7A1*, *CYP27A1*, *CYP8B1*, and *CYP7B1* [17], in addition to regulating bile acid [18] and drug metabolism [19].

Macrophage inhibitory cytokine 1 (MIC-1), also known as growth/differentiation factor 15, placental bone morphogenetic protein, nonsteroidal anti-inflammatory drug–activated gene 1, and placental transforming growth factor B, is a divergent member of the transforming growth factor-beta family that displays increased expression in response to macrophage activation [20]. MIC-1 is a stress-responsive cytokine generally expressed at high levels in various cancer types [21, 22], in rheumatoid arthritis, and during cardiovascular events [23, 24]. Elevated serum MIC-1 levels disrupt physiological appetite control pathways, causing anorexia, cachexia syndrome, and decreased body mass index [21, 22]. Serum MIC-1 levels increase with worsening disease stage and severity and can be used for disease diagnosis and prognostic prediction [21, 25]. MIC-1 acts via specific binding with glial-derived neurotrophic factor family receptor alpha-like (GFRAL) [26, 27].

Previous reports have suggested a relationship between cancer and cholecystic disease. In patients with cancer, the relative risk of cholecystitis is 1.38 (95% confidence interval 1.20–1.58) compared with the relative risk for the general population, and this risk doubles during the first 6 months following cancer diagnosis [28]. In addition, compared with patients without cancer, patients with pancreatic ductal adenocarcinoma were almost six times more likely to have experienced gallstone disease during the year prior to cancer diagnosis [29]. Therefore, cancer cells might secrete cytokines that promote gallstone formation. In this work, we examined the effects of MIC-1 on diet-induced gallstone formation.

## Materials and methods

### Materials

The following materials were used in this study: 1) MIC-1, the AMPK inhibitor Compound C, the AMPK activator AICAR (5-aminoimidazole-4-carboxamide-1-β-D-ribofuranosyl 5′-monophosphate), anti-CYP7A1 (SAB4301212) antibody, and anti-α-tubulin (T9026) antibody (Sigma-Aldrich, St. Louis, MO); 2) anti-phospho-JNK (Thr183/Tyr185) (9255), anti-phospho-ERK (Thr202/Tyr204) (4370), and anti-phospho-AMPK (Thr172) (2535) antibodies (Cell Signaling Technology, Beverly, MA); 3) anti-SREBP2 (NV100-74543) antibody (Novus Biologicals, Littleton, CO); 4) anti-CYP27A1 antibody (GTX103718) (GeneTex, San Antonio, TX); 5) anti-HMGCR antibody (ab174830) (Abcam, Cambridge, MA); 6) anti-bile salt export pump (BSEP; ABCB11) (sc-74500), anti-multidrug resistance 2 (MDR2; ABCB4) (sc-58221), anti-ATP-binding cassette transporter (ABCG) 5 (sc-517207), and anti-ABCG8 (sc-30111) antibodies (Santa Cruz Biotechnology, Santa Cruz, CA); and 7) anti-mouse-HRP (horseradish peroxidase) (115-036-003) and anti-rabbit-HRP (111-035-003) antibodies (Jackson Laboratory, Bar Harbor, ME).

### Animals and lithogenic diet feeding

Male C57BL/6J mice (6 weeks old) were purchased from Orient Bio, Inc. (Seoul, Korea) and housed under specific pathogen-free conditions. Experimental procedures were approved by the Animal Ethics Committee at LeeGilYa Cancer and Diabetes Institutes of Gachon University (LCDI-2018-0122). Mice were fed a lithogenic diet (D12336; Research Diets Inc., New Brunswick, NJ) and injected with either MIC-1 (200 μg/kg/week) or PBS for 6 weeks. Mice were euthanized with CO_2_. Sera, livers, small intestines, muscles (gastrocnemius), perigonadal adipose tissues, and gallbladders were collected and stored at -80°C until further analysis.

### Cell culture and MIC-1 treatment

Hep3B cells, C2C12 cells, 3T3-L1 cells, and Caco2 cells were obtained from the Korean Cell Line Bank (Seoul National University, Republic of Korea) and grown in Dulbecco’s modified Eagle medium (HyClone, Logan, UT), supplemented with 10% fetal bovine serum and 1% penicillin/streptomycin (HyClone). For the cell experiments, 5, 10, 20 ng/ml MIC-1 was used.

### Cholesterol, phospholipid, and bile acid measurements

Cholesterol, phospholipid, and bile acid levels in sera, bile, feces, and livers were measured using the Total Cholesterol and Cholesteryl Ester Colorimetric/Fluorometric Assay Kit (BioVision, Mountain View, CA), the Phospholipid Assay Kit (Sigma-Aldrich), and the Total Bile Acids Assay Kit (BioVision), respectively, according to the corresponding manufacturers’ protocols.

### Western blotting

Hep3B cells, C2C12 cells, 3T3-L1 cells, or liver samples were homogenized using RIPA buffer (50 mM Tris-Cl, pH 7.5; 150 mM NaCl, 1% Nonidet P-40, 0.5% sodium deoxycholate, 0.1% SDS, protease and phosphatase inhibitors (Sigma-Aldrich)) and kept for 30 min at 4°C. After centrifugation (10,000×g, 10 min, 4°C), protein concentrations were measured, and 50 μg of proteins were separated on 8~15% SDS polyacrylamide gels and further transferred to nitrocellulose (NC) membranes (Bio-Rad Laboratories, Hercules, CA). NC membranes were blocked with 5% bovine serum albumin (Sigma-Aldrich) in TBST (TBS with 0.1% Tween 20) for 1 h and incubated with primary antibodies overnight at 4°C. The next day, secondary antibodies were attached for 1 h at room temperature. Protein bands were detected by the ChemiDoc MP imaging system (Bio-Rad Laboratories), using EzWestLumi Plus Reagents (ATTO Corporation, Tokyo, Japan).

### CYP7A1, ABCG5, ABCG8 luciferase reporter assay

The Dual-Luciferase Reporter assay system (Promega, San Luis Obispo, CA) was used for the luciferase activity assay. A reporter plasmid containing the CYP7A1 promoter region was kindly provided by Professor John Y.L. Chiang (Department of Biochemistry and Molecular Pathology, Northeastern Ohio Universities College of Medicine, USA) [30] and reporter plasmids containing ABCG5 and ABCG8 promoter regions were generated according to a previous study [6]. These plasmids were transfected into Hep3B cells using Lipidofect-P transfection reagent (Lipidomia, Seongnam, Republic of Korea). *Renilla* luciferase vector was used for the normalization of the transfection efficiency. After 24 h of transfection, 10 ng/ml MIC-1 or 10 μM Compound C were treated and incubated for another 12 h. Firefly and *Renilla* luciferase activities were measured using a GloMax^™^ 20/20 Luminometer (Promega).

### Real-time PCR

Total mRNA from the liver, small intestine, or Caco2 cells was extracted using RNAiso Plus (Takara, Shiga, Japan), and cDNA was immediately synthesized using PrimeScript^™^ RT Reagent Kit with gDNA Eraser (Takara). Real-time PCR was performed using the SYBR^®^ Premix Ex Taq^™^ II, ROX Plus (Takara) on a Bio-Rad CFX96 system (Bio-Rad Laboratories). Relative gene expression was calculated by using the 2^−ΔΔCt^ method [31]. The primers used are described in Table 1.

**Table 1:** Primers used for real-time PCR.

Gene	Primer Sequences	Reference
*SREBP2*	F: 5′-ACTGACCAGCACCCATACTC-3′	[32]
(mouse)	R: 5′-CAGGAGGAGAGTTGGAACCA-3′	
*HMGCR*	F: 5′-ATCTCCTCTCCACAAAGCTT-3′	[32]
(mouse)	R: 5′-CATTCTCACAGCAAGCTCCC-3′	
*CYP7A1*	F: 5′-CTCCGGGCCTTCCTAAATCA-3′	[32]
(mouse)	R: 5′-ACAGCGTTAGATATCCGGCT-3′	
*CYP7B1*	F: 5′-GCATCATCCGAGAAGTGCAG-3′	[32]
(mouse)	R: 5′-ATGAGTGGAGGAAAGAGGGC-3′	
*CYP27A1*	F: 5′-GAGAGTGAATCAGGGGACCA-3′	[32]
(mouse)	R: 5′-TCAGGAATGGAGGGTTTCAG-3′	
*CYP8B1*	F: 5′-AGTTGCAGCGTCTCTTCCAT-3′	[32]
(mouse)	R: 5′-CCTTGCTCCCTCAGAAACTG-3′	
*SHP* (*NR0B2*)	F: 5′-AGCTGGGTCCCAAGGAGTAT-3′	[32]
(mouse)	R: 5′-GGTACCAGGGCTCCAAGACT-3′	
*FXR* (*NR1H4*)	F: 5′-TGGGTACCAGGGAGAGACTG-3′	[32]
(mouse)	R: 5′-GTGAGCGCGTTGTAGTGGTA-3′	
*PXR* (*NR1I2*)	F: 5′-CCCATCAACGTAGAGGAGGA-3′	[32]
(mouse)	R: 5′-TCTGAAAAACCCCTTGCATC-3′	
*ABCG5*	F: 5′-AATTTTGGGGGAATTTCCAG-3′	[32]
(mouse)	R: 5′-GTCCTGTGGTTGGCTCATCT-3′	
*ABCG8*	F: 5′-CCTGATCCGTCGTCAGATTT-3′	[32]
(mouse)	R: 5′-CCATGGCCGTAGTAAAGGAA-3′	
*BSEP* (*ABCB11*)	F: 5′-GGGTTCTACAGGGGTTGGAA-3′	[32]
(mouse)	R: 5′-GTGAACTTGGCCACACTCAG-3′	
*MDR2* (*ABCB4*)	F: 5′-TCGCAGAGAACATCGCCTAT-3′	[32]
(mouse)	R: 5′-TCTCGATGAAGGGGTGGATG-3′	
*LXRα* (*NR1H3*)	F: 5′-TACGTCTCCATCAACCACCC-3′	[32]
(mouse)	R: 5′-CTTGCTCTGAATGGACGCTG-3′	
*LXRβ* (*NR1H2*)	F: 5′-CAGACGCTACAACCACGAGA-3′	[32]
(mouse)	R: 5′-ATGAATTCCACCTGCAAGCC-3′	
*LRH1*	F: 5′-TGTCCAAGAGCAGGTGAATG-3′	
(mouse)	R: 5′-TCGGGTAGCCGAAGAAGTAG-3′	
*HNF4α*	F: 5′-GGATTACATCAACGACCGGC-3′	
(mouse)	R: 5′-TTCGATCATCTGCCAGGTGA-3′	
*NR1I3* (*CAR*)	F: 5′-TGCCTAAGGGAAACAGGAGA-3′	
(mouse)	R: 5′-GGTCTCCACACACCACACAG-3′	
*NPC1L1*	F: 5′-ATCGCACTACCATCCAGGACCT-3′	[33]
(mouse)	R: 5′-CCCAGAGTAGCCTTGGAATCCA-3′	
*GAPDH*	F: 5′-ACTCACGGCAAATTCAACGG-3′	
(mouse)	R: 5′-ATGTTAGTGGGGTCTCGCTC-3′	
*ABCG5*	F: 5′-AATGACTGCGGTTACCCTG-3’	
*(human)*	R: 5′-TTCTATTTCCCGTTCCTTGC-3′	
*ABCG8*	F: 5′-GTTCATGATCGGTGCTCTCA-3’	
*(human)*	R: 5′-GCCCGTCTTCCAGTTCATAG-3′	
*NPC1L1*	F: 5′-GGGTGGATGACTTCATTGACTGG-3’	[34]
*(human)*	R: 5′-CATCGTGATGCTCATGCAGTTC-3′	
*GAPDH*	F: 5′-ACACCCACTCCTCCACCTTT-3’	
*(human)*	R: 5′-TGCTGTAGCCAAATTCGTTG-3′	

SREBP2, sterol regulatory element binding protein-2; HMGCR: HMG-CoA reductase; CYP7B1, oxysterol 7α-hydroxylase; CYP7A1, cholesterol 7α-hydroxylase; CYP27A1, sterol 27-hydroxylase; CYP8B1, sterol 12α-hydroxylase; BSEP, bile salt export pump; MDR2, multidrug resistance 2; NPC1L1, Niemann-Pick C1-Like 1; ABCG, ATP-binding cassette transporter; FXR, farnesoid X receptor; PXR, pregnane X receptor; SHP, small heterodimer partner; LXR, liver X receptor; LRH1, liver receptor homolog-1; HNF4α, hepatocyte nuclear factor 4α; CAR, constitutive androstane receptor; GAPDH, glyceraldehyde 3-phosphate dehydrogenase.

### Statistical analyses

All experiments were repeated at least three times independently, and values are presented as means ± standard error of the mean. Statistical significance was calculated using analysis of variance (ANOVA), followed by Tukey’s post hoc test (GraphPad Prism 6.0; GraphPad Software, San Diego, CA).

## Results

### MIC-1 aggravates diet-induced gallstone formation

**Fig 1:**
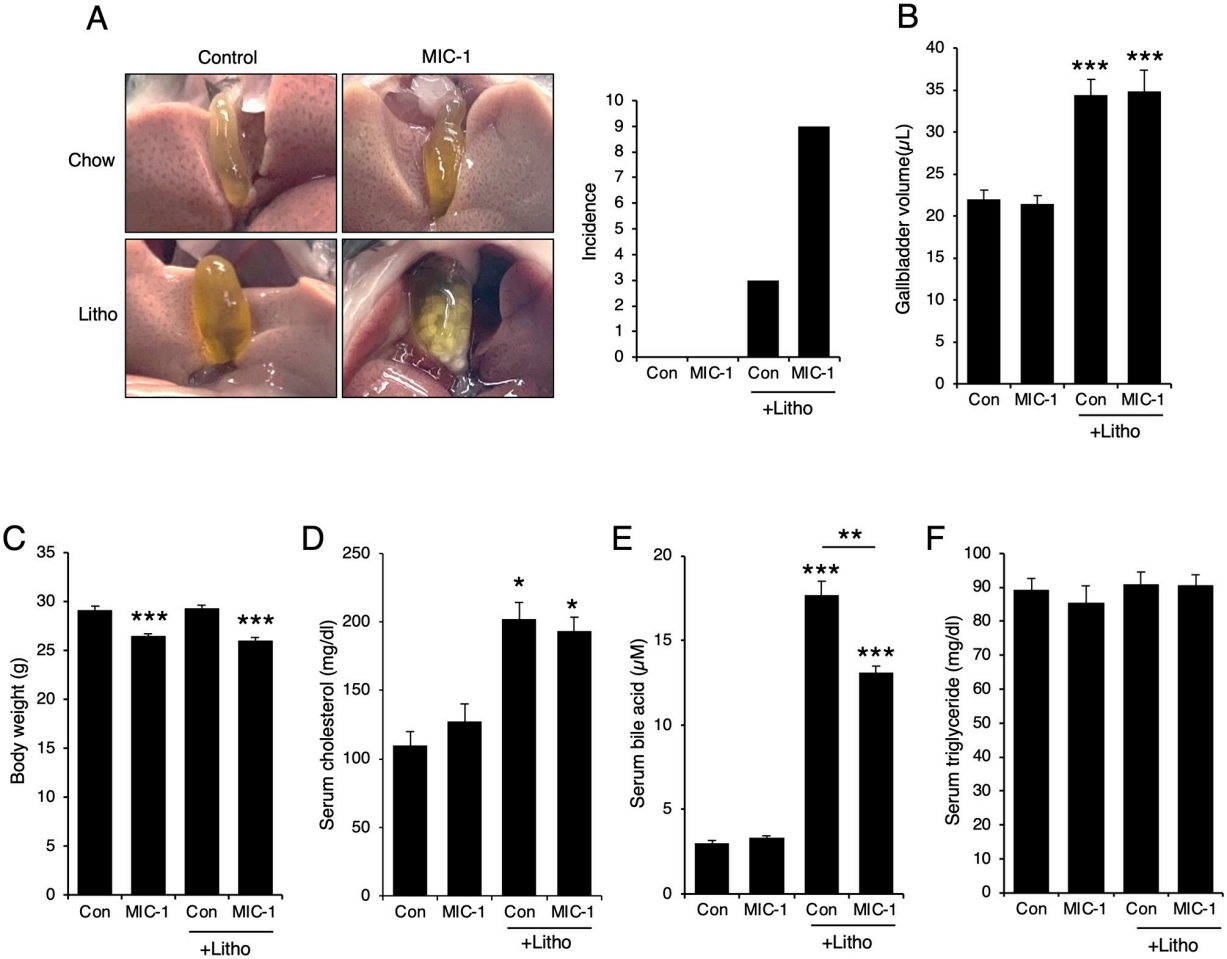
MIC-1 treatment increases diet-induced gallstone formation. C57BL/6J mice were fed a lithogenic diet for 6 weeks and intraperitoneally administered macrophage inhibitory cytokine 1 (MIC-1; 200 μg/kg/week) or phosphate-buffered saline (PBS). (**A**) Images showing gallstone formation (left panel) and incidence (right panel; n = 10). (**B**) Gallbladder volumes and (**C**) body weights after 6 weeks on lithogenic diet (n = 10). Serum cholesterol (**D**), bile acid (**E**), and triglyceride (**F**) levels were measured with commercial colorimetric kits (n = 8). Values are presented as the mean ± standard error of the mean (SEM). *p<0.05, **p<0.01, ***p<0.001. Three independent experiments were performed.

C57BL/6 mice were fed a lithogenic diet for 6 weeks, resulting in 3 of 10 mice exhibiting gallstone formation. When lithogenic diet–fed mice were also treated with MIC-1, 9 of 10 mice exhibited gallstone formation (Fig 1A). By contrast, mice fed with standard chow did not show any gallstone formation (Fig 1A). Gallbladder volumes increased in mice fed a lithogenic diet compared with mice fed standard chow, but no differences in gallbladder volumes were observed between lithogenic diet–fed mice treated with MIC-1 and those treated with PBS (Fig 1B). The body weights of MIC-1–treated mice were reduced compared with PBS-treated mice (Fig 1C). Serum cholesterol and bile acid levels increased in mice fed lithogenic diet compared with those in mice fed standard chow. Among lithogenic diet–fed mice, serum bile acid levels decreased in MIC-1–treated mice compared with PBS-treated mice (Fig 1D and 1E), whereas serum triglyceride levels were not affected (Fig 1F).

### MIC-1 reduces both cholesterol and bile acid synthesis

**Fig 2:**
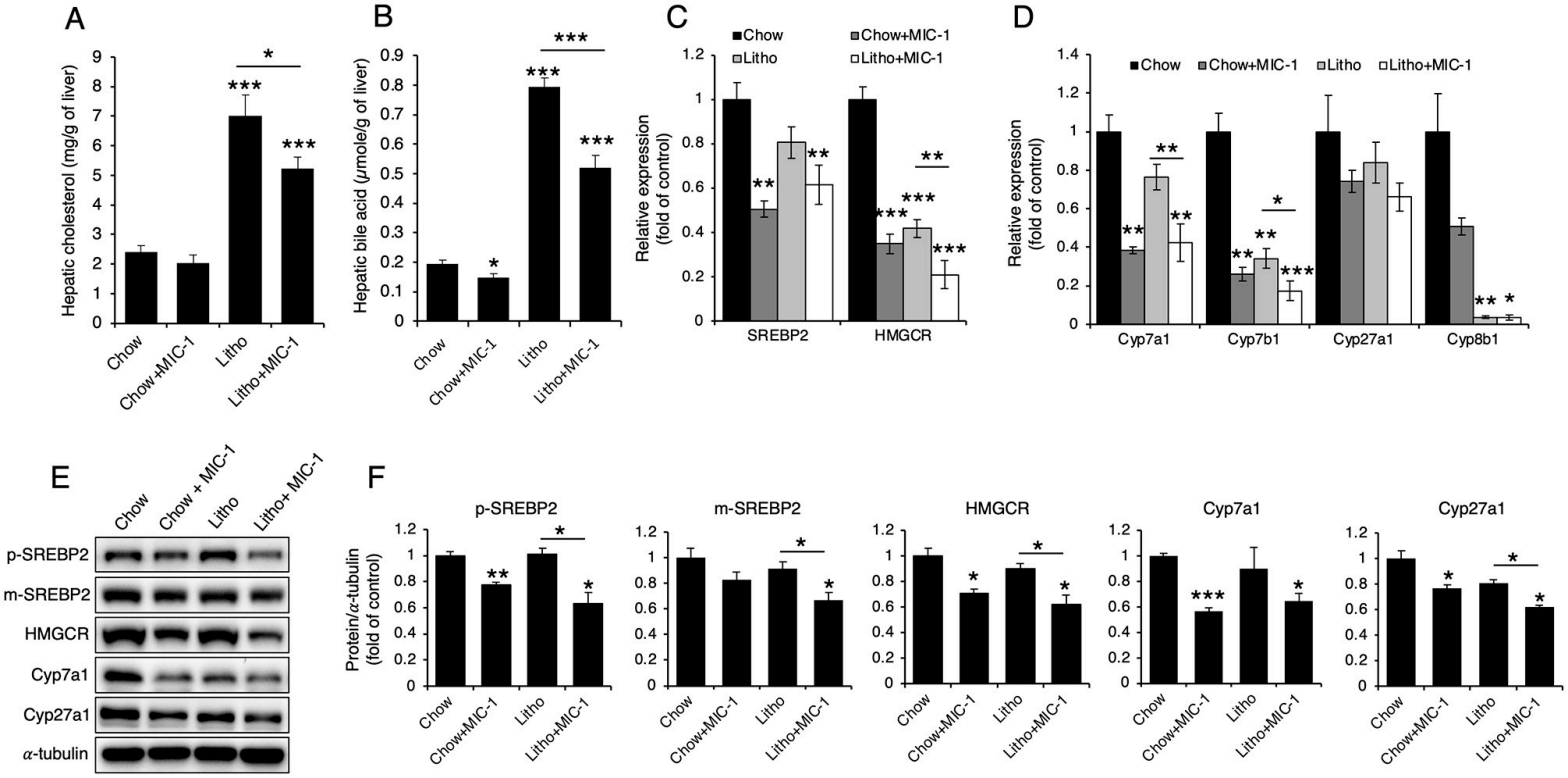
MIC-1 reduces cholesterol and bile acid synthesis. (**A**) Total hepatic cholesterol and (**B**) total hepatic bile acid levels in mice treated with MIC-1 were quantified using commercial kits (n = 8). (**C** and **D**) Real-time PCR assessment of (**C**) *SREBP2* and *HMGCR* mRNA levels and (**D**) *CYP7A1*, *CYP7B1*, *CYP27A1*, and *CYP8B1* mRNA levels in the livers of mice treated with MIC-1 (n = 5). Representative western blots (**E**) and associated densitometric analyses (**F**) of the levels of precursor (p) and mature (m) forms of SREBP2, HMGCR, CYP7A1, and CYP27A1 (n = 3). Values are presented as the mean ± SEM. *p<0.05, **p<0.01, ***p<0.001. Three independent experiments were performed.

Because lithogenic diet–fed mice treated with MIC-1 presented with decreased serum bile acid levels relative to mice treated with PBS (Fig 1D), we further analyzed whether MIC-1 treatment affected hepatic cholesterol and bile acid levels. Lithogenic diet–fed mice showed increased hepatic cholesterol and bile acid levels compared with standard chow-fed mice, and among lithogenic diet–fed mice, MIC-1 treatment reduced both cholesterol and bile acid levels compared with PBS treatment (Fig 2A and 2B). Because SREBP2 and HMGCR are the primary regulators of cholesterol synthesis [11, 12], we next measured their expression levels following MIC-1 treatment. The expression levels of both hepatic SREBP2 and HMGCR decreased in lithogenic diet–fed mice treated with MIC-1 compared with mice treated with PBS (Fig 2C, 2E and 2F). We also measured expression levels of enzymes involved in bile acid synthesis [16] and found decreased expression levels of CYP7A1, CYP7B1, and CYP27A1 but not CYP8B1 in mice treated with MIC-1 compared with mice treated with PBS (Fig 2D–2F).

### MIC-1 reduces CYP7A1 and HMGCR expression via AMPK activation

**Fig 3:**
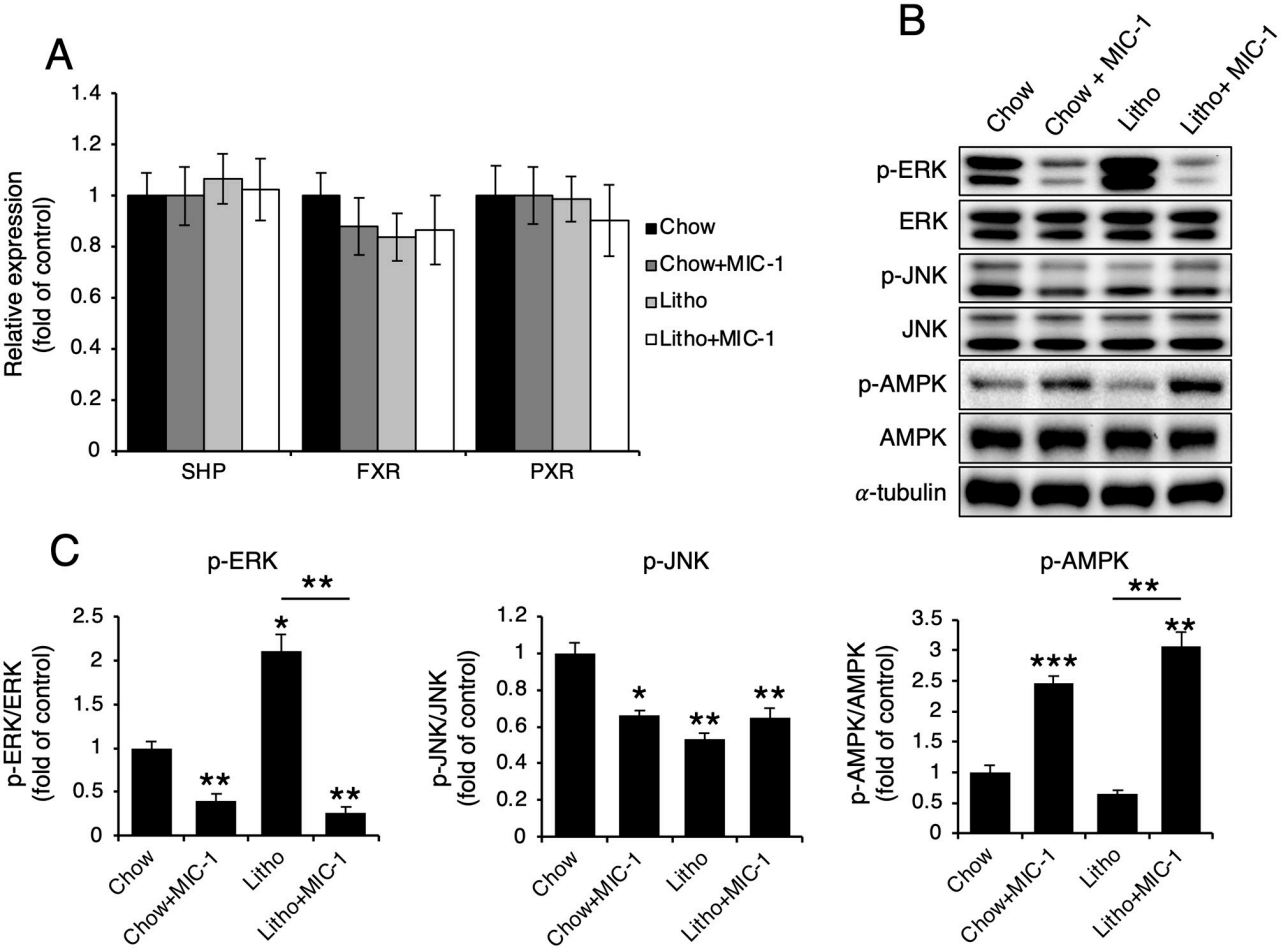
MIC-1 increases AMPK phosphorylation. (**A**) Real-time PCR measurements of *SHP*, *FXR*, and *PXR* mRNA levels in the livers of mice treated with MIC-1 (n = 5). (**B**) Representative western blots of the phosphorylated forms of ERK, JNK, and AMPK and (**C**) their densitometric analyses (n = 3). Values are presented as the mean ± SEM. Three independent experiments were performed.

SHP, FXR, PXR, extracellular signal–related kinase (ERK), and c-Jun N-terminal kinase (JNK) are well-known regulators of CYP7A1 [16]; therefore, we examined the expression levels of these regulators and found that SHP, FXR, and PXR expression did not differ between mice treated with MIC-1 and those treated with PBS (Fig 3A). In addition, ERK and JNK phosphorylation decreased in mice treated with MIC-1 compared with mice treated with PBS (Fig 3B and 3C), indicating that these signaling pathways are not involved in the observed MIC-1-induced reduction in CYP7A1 expression. However, AMPK phosphorylation increased in MIC-1–treated mice compared with PBS-treated mice (Fig 3B and 3C).

**Fig 4:**
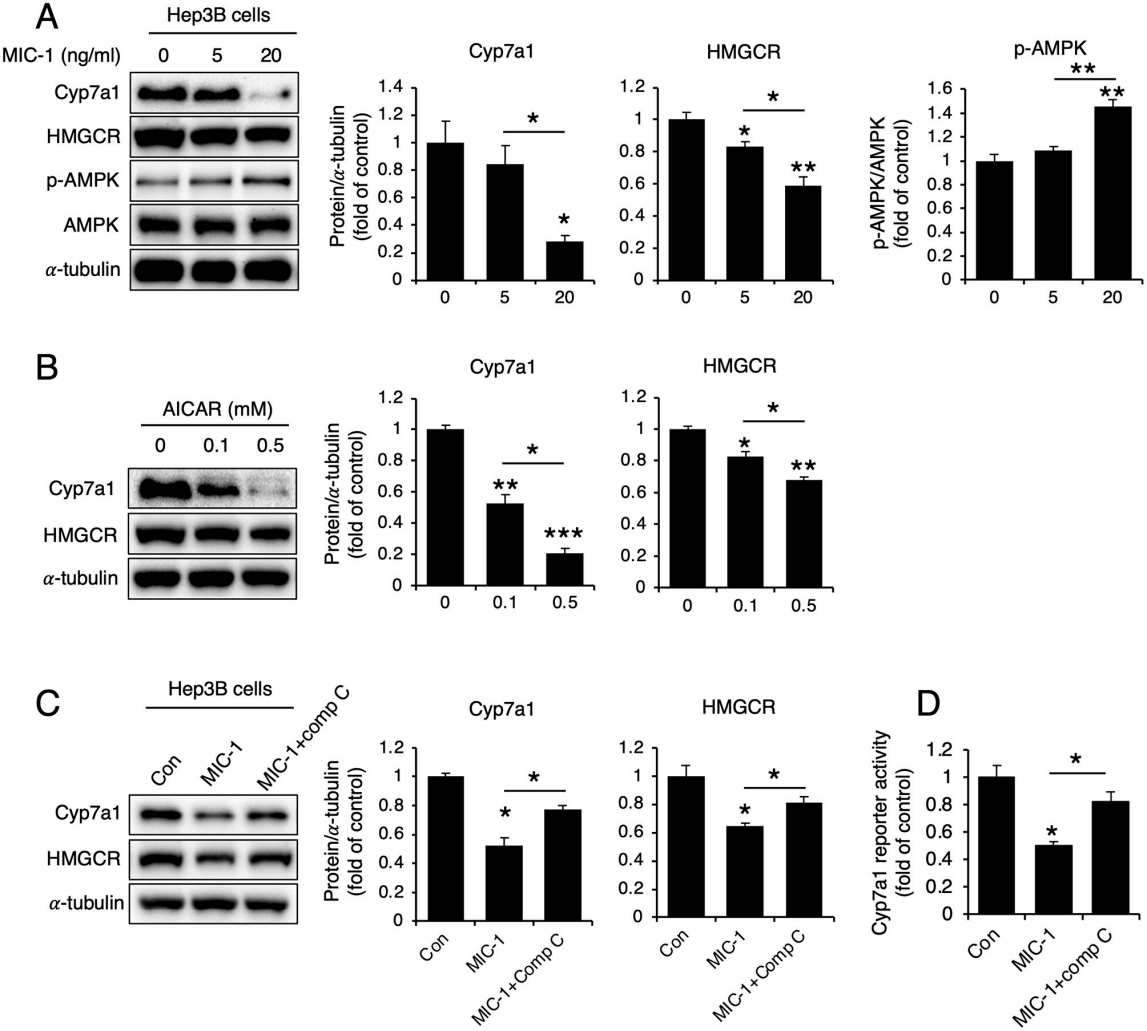
AMPK phosphorylation due to MIC-1 plays a critical role in CYP7A1 and HMGCR downregulation. Representative western blots and their densitometric analyses showing cholesterol 7α-hydroxylase (CYP7A1) and 3-hydroxy-3-methylglutaryl-CoA reductase (HMGCR) expression levels in (**A**) Hep3B cells treated with 5 or 20 ng/ml MIC-1, (**B**) Hep3B cells treated with 0.1 or 0.5 mM 5-aminoimidazole-4-carboxamide ribonucleoside (AICAR; AMP-activated protein kinase [AMPK] activator), and (**C**) Hep3B cells co-treated with 20 ng/ml macrophage inhibitory cytokine 1 (MIC-1) and 10 μM Compound C (Comp C; AMPK inhibitor) (n = 3). (**D**) CYP7A1 reporter luciferase activity assay after co-treatment with 20 ng/ml MIC-1 and 10 μM Comp C (AMPK inhibitor) (n = 3). Values are presented as the mean ± SEM. *p<0.05. Three independent experiments were performed.

To further examine whether AMPK affects CYP7A1 and HMGCR expression, we treated Hep3B cells with MIC-1. AMPK phosphorylation increased in cells treated with MIC-1 compared with control cells, and the expression levels of both CYP7A1 and HMGCR decreased (Fig 4A). Treatment of Hep3B cells with the AMPK activator 5-aminoimidazole-4-carboxamide ribonucleoside (AICAR) decreased both CYP7A1 and HMGCR levels (Fig 4B), whereas treatment with the AMPK inhibitor Compound C reversed MIC-1-induced CYP7A1 and HMGCR downregulation (Fig 4C). Similarly, MIC-1 treatment reduced CYP7A1 luciferase activity in Hep3B cells, which was reversed by Compound C treatment (Fig 4D).

### MIC-1 increases ABCG5/8 expression

**Fig 5:**
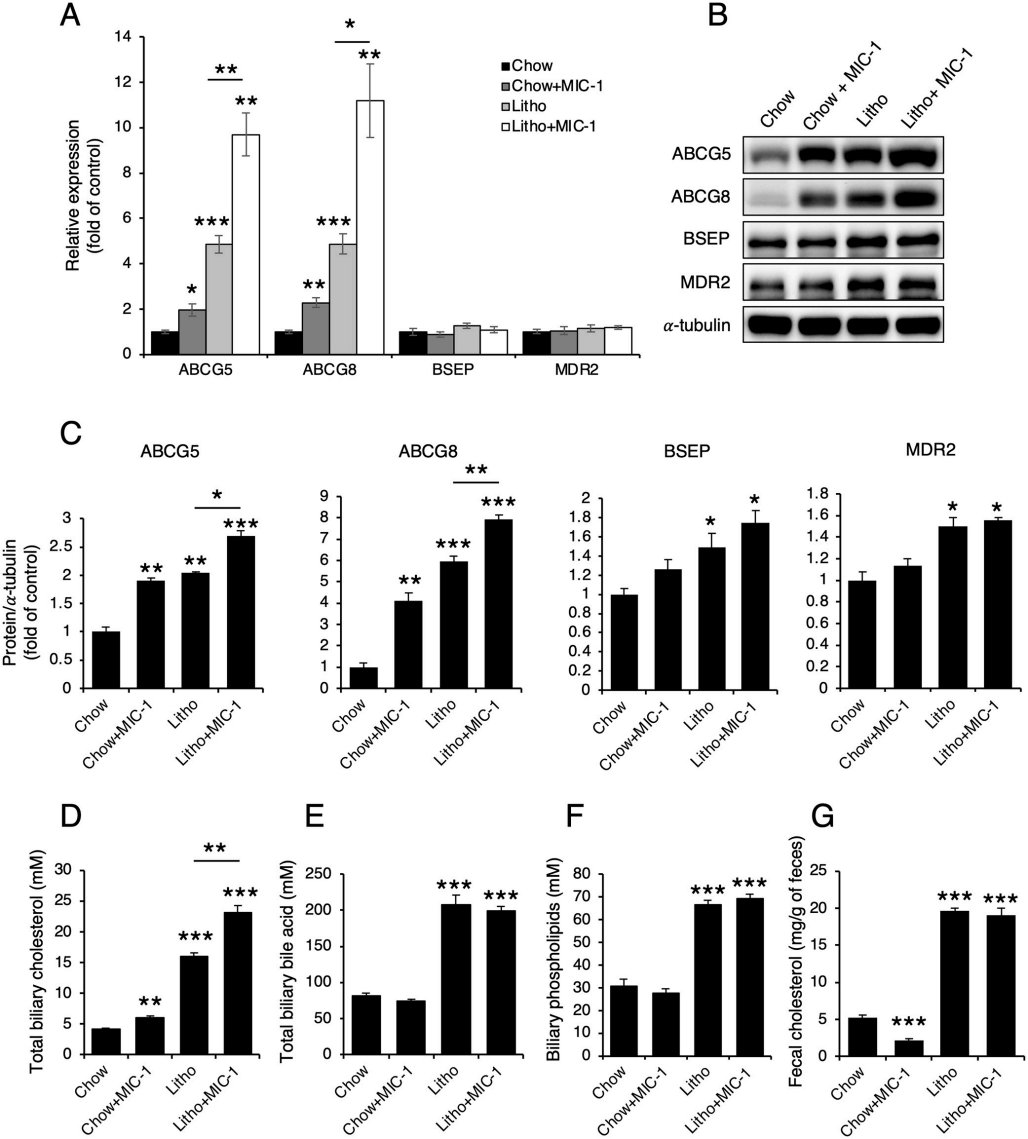
MIC-1 increases cholesterol secretion through ABCG5 and ABCG8 overexpression. ATB-binding cassette subfamily G (ABCG)5, ABCG8, bile salt export pump (BSEP), and multidrug resistance protein 2 (MDR2) expression in the livers of mice treated with macrophage inhibitory cytokine 1 (MIC-1). (**A**) mRNA levels (n = 5). (**B**) Representative western blots and (**C**) their densitometric analyses (n = 3). Biliary cholesterol (**D; n = 5**), biliary bile acid (**E; n = 5**), biliary phospholipid (**F; n = 5**), and fecal cholesterol (**G; n = 6**) levels were measured in mice treated with MIC-1. Values are presented as the mean ± SEM. *p<0.05, **p<0.01, ***p<0.001. Three independent experiments were performed.

Gallstone formation is regulated by the balance between biliary cholesterol, biliary phospholipids, and bile acids, the secretion of which are regulated by ABCG5/8 heterodimers, bile salt export pump (BSEP, also known as ABC subfamily B [ABCB]11), and multidrug resistance protein 2 (MDR2, also known as ABCB4), respectively [1]. Therefore, we examined the expression levels of ABCG5/8, BSEP (ABCB11), and MDR2 (ABCB4). Compared with PBS treatment, MIC-1 treatment increased the expression levels of ABCG5 and ABCG8 but not BSEP or MDR2 (Fig 5A–5C). As expected, MIC-1 treatment increased biliary cholesterol levels (Fig 5D) but not biliary bile acid or biliary phospholipid levels (Fig 5E and 5F) compared with PBS treatment. Furthermore, among standard chow–fed mice, fecal cholesterol excretion was reduced following MIC-1 treatment compared with PBS treatment, but this effect was not observed in MIC-1–treated mice fed lithogenic diet (Fig 5G).

**Fig 6:**
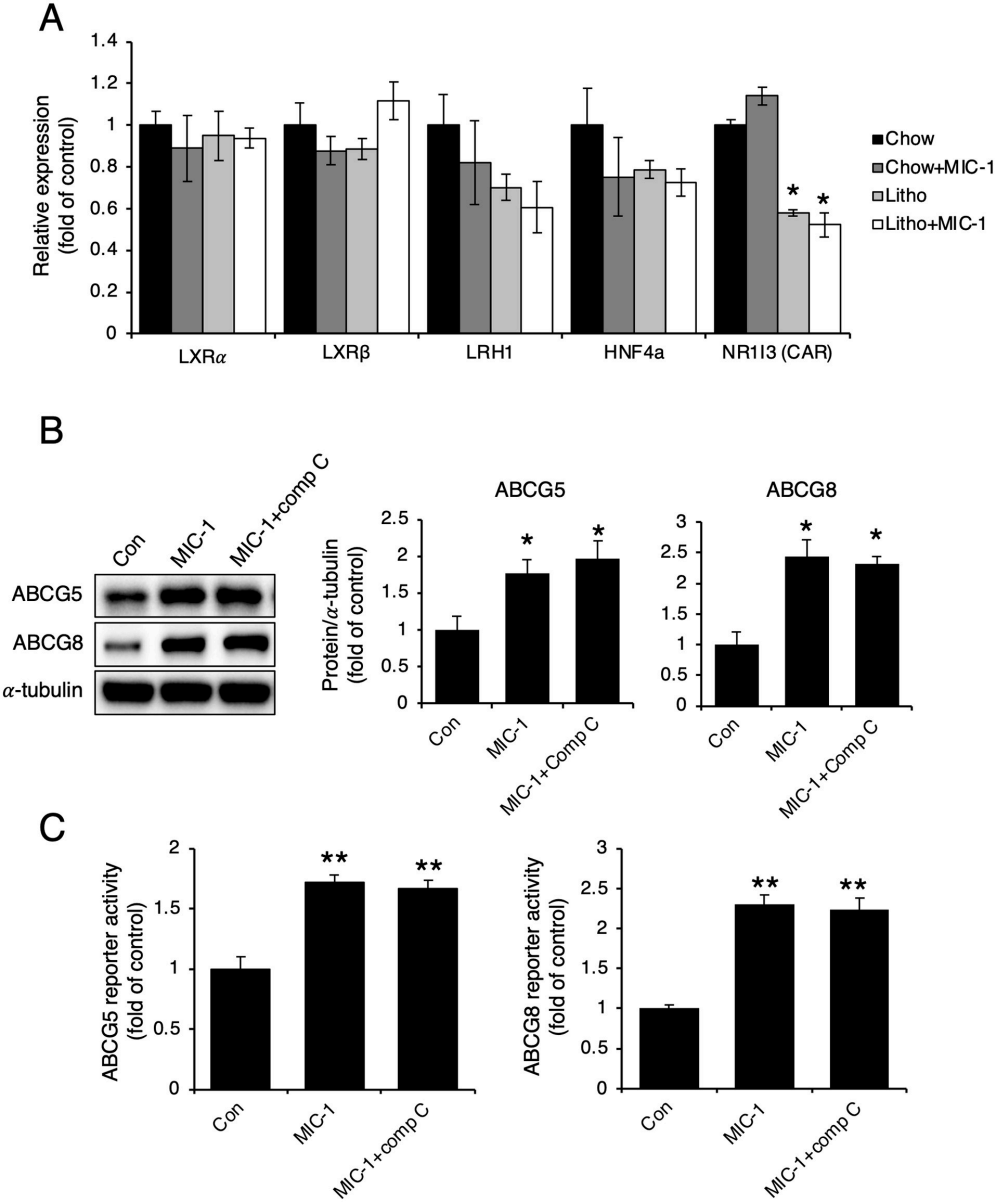
MIC-1 plays an important role in the upregulation of ABCG5 and ABCG8. (**A**) Real-time PCR measurements of *LXRα/β*, *LRH1*, *HNF4α*, and *NR1I3* mRNA levels in the livers of mice treated with macrophage inhibitory cytokine 1 (MIC-1; n = 5). (**B**) Representative western blots and their densitometric analyses showing ATP-binding cassette subfamily G (ABCG)5 and ABCG8 expression levels in Hep3B cells co-treated with 20 ng/ml MIC-1 and 10 μM Compound C (comp C; AMPK inhibitor; n = 3). (C) ABCG5 and ABCG8 reporter activities in Hep3B cells co-treated with 20 ng/ml MIC-1 and 10 μM Compound C (comp C; AMPK inhibitor; n = 3). Values are presented as the mean ± SEM. **p<0.01. Three independent experiments were performed.

We next examined LXRα/β, LRH1, HNF4α, and NR1I3 levels because these nuclear receptors are known to regulate ABCG5/8 heterodimers [4, 6–8]. However, LXRα/β, LRH1, HNF4α, and NR1I3 expression levels did not differ between PBS-treated and MIC-1–treated mice (Fig 6A), indicating that these proteins are not involved in the observed MIC-1–induced increases in ABCG5/8 expression. Because AMPK phosphorylation was elevated in MIC-1–treated Hep3B cells, we co-treated Hep3B cells with MIC-1 and the AMPK inhibitor Compound C. Although MIC-1 treatment increased ABCG5/8 protein levels and promoter activities, Compound C had no effects on ABCG5/8 protein levels or promoter activities, indicating that the observed MIC-1–induced changes in ABCG5/8 expression did not involve AMPK signaling (Fig 6B and 6C).

## Discussion

Gallstone disease is common, diagnosed in 10%–15% of adults of European descent and in 5% of adults of Asian descent [2]. More than 80% of patients with gallstones are asymptomatic and unaware of the presence of gallstones [35]. Gallstone formation can be prevented by reducing biliary cholesterol secretion (through the downregulation of ABCG5/8) or increasing FXR expression, which increases biliary bile acid and phospholipid secretion (via BSEP and MDR2) [36]. Therefore, ABCG5/8 and FXR represent potentially important therapeutic targets for gallstone disease. In this study, we found that MIC-1 treatment induces gallstone formation by increasing ABCG5/8 expression and altering cholesterol and bile acid homeostasis.

Compared with the livers of PBS-treated mice, the livers of MIC-1–treated mice showed reduced levels of CYP7A1 and HMGCR, which are the rate-limiting enzymes for bile acid and cholesterol synthesis, respectively [10, 15]. Because MIC-1 treatment reduces food intake and inhibits weight gain [37], the energy sensor AMPK may be activated in response to MIC-1 treatment [13]. Our study showed that MIC-1 treatment in Hep3B, C2C12, and 3T3-L1 cells activated AMPK phosphorylation (S1 Fig), suggesting that MIC-1 treatment may directly activate AMPK. AMPK regulates many important pathways, including glucose uptake, fatty acid uptake, fatty acid synthesis, cholesterol synthesis, glycogen synthesis, and protein synthesis [13]. AMPK also regulates SREBP2, HMGCR [38, 39], and CYP7A1 [40]. Furthermore, both glucose and the activation of p70 ribosomal S6 kinase induce CYP7A1 transcription by reducing AMPK signaling [40, 41], indicating that energy status could play an important role in CYP7A1 expression. In our study, MIC-1 not only reduced body weight but also increased AMPK phosphorylation, which affected CYP7A1 and HMGCR expression. Although MIC-1 treatment reduced hepatic cholesterol levels, no effects were observed on the cholesterol levels in skeletal muscle and adipose tissues (S2 Fig), suggesting that the cholesterol-lowering effects of MIC-1 may be limited to the liver.

Although AMPK is known to regulate ABCG5/8 expression [42], the MIC-1 treatment-induced increases in ABCG5/8 levels did not involve AMPK activation, as treatment with the AMPK inhibitor Compound C did not reverse the MIC-1-induced overexpression of ABCG5/8. Although LXRα/β [4], LRH1 [6], HNF4α [7], and NR1I3 [8] are able to regulate ABCG5/8 expression, the levels of these regulators were not altered by MIC-1 treatment. MIC-1 treatment appeared to alter ABCG5/8 reporter activities, suggesting that MIC-1 can directly regulate ABCG5/8 expression.

MIC-1 treatment increased hepatic ABCG5/8 protein levels but reduced fecal cholesterol excretion. The intestinal expression levels of ABCG5/8 and NPC1L1 play important roles in cholesterol absorption and secretion [33], and ABCG5/8 overexpression increases fecal cholesterol excretion [5]. Because MIC-1 reduces hepatic cholesterol synthesis by decreasing HMGCR levels, intestinal cholesterol absorption might increase to maintain the total cholesterol level in the liver. Indeed, our study showed elevated NPC1L1 expression in the small intestines of MIC-1–treated mice compared with PBS-treated mice (S3A Fig). A previous study reported that the inhibitor of cholesterol synthesis atorvastatin also increased intestinal NPC1L1 expression [43], leading to increased cholesterol absorption. Similarly, intestinal NPC1L1 expression was increased for lithogenic diet–fed mice treated with MIC-1 compared with PBS-treat mice (S3A Fig). However, MIC-1 treatment of Caco2 cells demonstrated no effects on ABCG5/8 or NPC1L1 expression (S3B Fig). The observed increase in NPC1L1 expression among MIC-1–treated mice was not a direct effect of MIC-1 treatment but may be associated with the MIC-1–induced decrease in hepatic cholesterol. Therefore, our findings suggest that MIC-1 treatment only affects hepatic ABCG5/8 expression without affecting intestinal ABCG5/8 or NPC1L1 expression.

Cholecystitis and gallstones are more common in patients with cancer than in those without cancer [28], but the involvement of MIC-1 in these phenomena remains unknown. MIC-1 is a pro-cachectic factor that causes abrupt weight loss, a known risk factor for gallstone formation. In addition to weight loss, our study revealed that MIC-1 contributes to gallstone formation through ABCG5/8 overexpression. However, the role played by MIC-1 in gallstone formation in patients with cancer requires additional clinical validation.

A recent report indicated that long-acting MIC-1 molecules could be used for obesity treatment [44]. MIC-1 binds GFRAL and regulates appetite and energy homeostasis [22]. MIC-1 treatment reduces body weight due to reduced energy intake, and MIC-1 transgenic mice are resistant to obesity and glucose intolerance [21, 37]. Rapid weight loss due to a low-calorie diet or bariatric surgery induces bile acid stasis and can lead to gallstone development [35]. Because MIC-1 treatment reduces food intake, MIC-1 treatment is associated with a rapid weight loss effect similar to that observed with a low-calorie diet. Therefore, MIC-1 treatment supports weight loss but can induce gallstone formation due to AMPK activation and ABCG5/8 overexpression. As a result, the potential induction of gallstone formation by MIC-1, mediated by AMPK activation and ABCG5/8 overexpression, should be monitored during obesity treatments using MIC-1.

## Supporting information

S1 Fig MIC-1 activates AMPK signaling in Hep3b, C2C12, and 3T3-L1 cells. Representative western blots (top) and their densitometric analyses (bottom) show expression of AMP-activated protein kinase (AMPK), phosphorylated AMPK (p-AMPK), and α-tubulin in (A) Hep3B, (B) C2C12, and (C) 3T3-L1 cells after treatment with 5 or 20 ng/ml macrophage inhibitory cytokine 1 (MIC-1) for 24 h (n = 3). Values are presented as the mean ± SEM. *p<0.05. Three independent experiments were performed.
(TIFF)

S2 Fig MIC-1 injection does not affect cholesterol levels in muscle and adipose tissues. Cholesterol levels were measured in muscle (A) and adipose tissues (B) obtained from mice injected with macrophage inhibitory cytokine 1 (MIC-1; n = 5). Values are presented as the mean ± SEM. Three independent experiments were performed. Chow, standard chow diet; Litho, lithogenic diet.
(TIFF)

S3 Fig MIC-1 injection does not affect intestinal ABCG5/8 and NPC1L1 expression. Real-time PCR was used to measure the mRNA levels of ATP-binding cassette superfamily G (*ABCG5*), *ABCG8*, and Niemann-Pick C1-like 1 (*NPC1L1*) in the (A) small intestines of mice injected with macrophage inhibitory cytokine 1 (MIC-1) (n = 5) and (B) Caco2 cells treated with 10 ng/ml MIC-1 for 24 h (n = 5). Values are presented as the mean ± SEM. *p<0.05, **p<0.01, ***p<0.001. Three independent experiments were performed. Chow, standard chow diet; Litho, lithogenic diet; Con, control.
(TIFF)

S1 Raw images (PDF)
