# Supplementary figures and images for: Macrophage inhibitory cytokine-1 aggravates diet-induced gallstone formation via increased ABCG5/ABCG8 expression
Source: PLoS One. 2023 Jun 13;18(6):e0287146. doi: 10.1371/journal.pone.0287146 (PMC10263326; doi:10.1371/journal.pone.0287146)

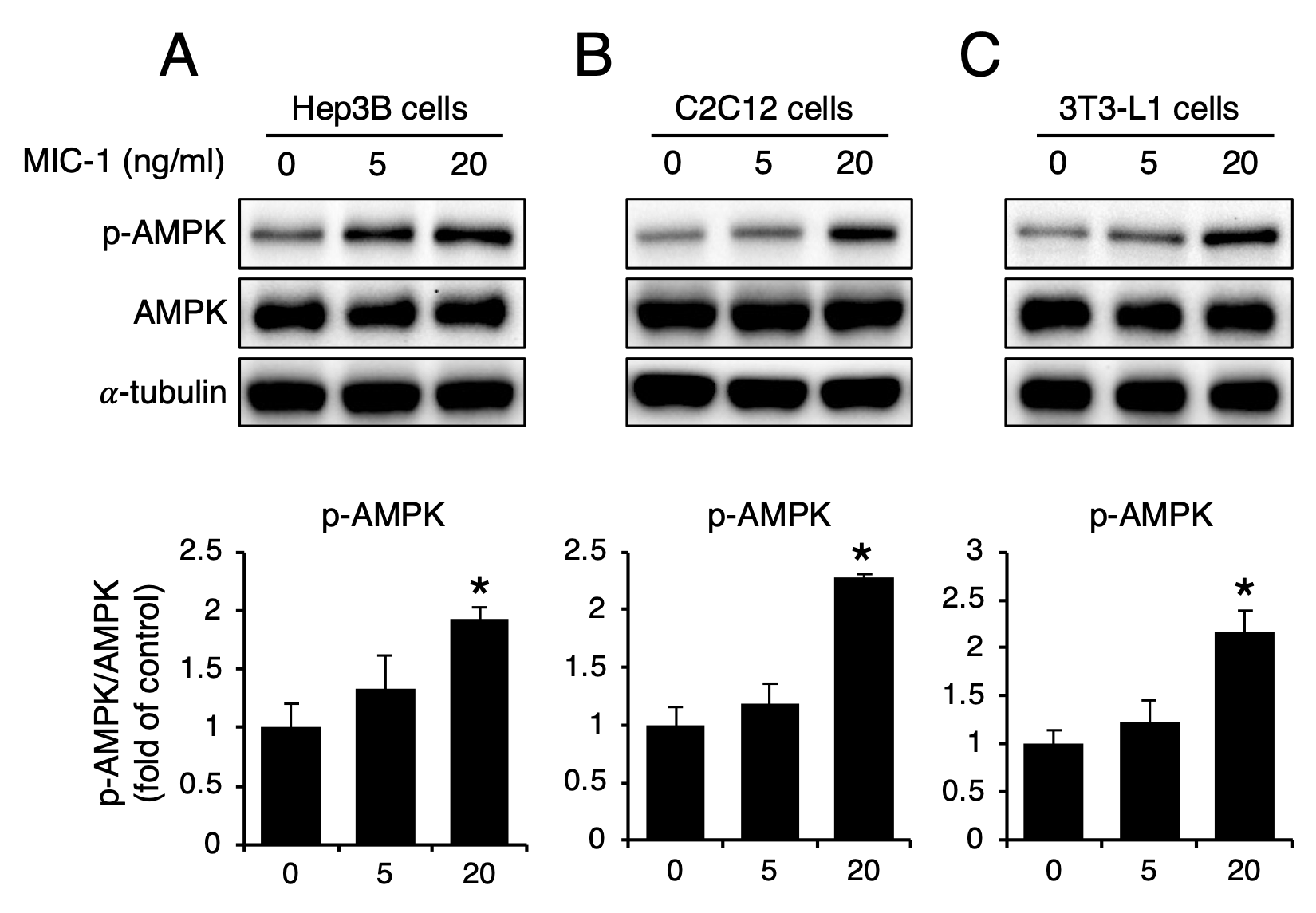

Supplement: S1 Fig — Representative western blots (top) and their densitometric analyses (bottom) show expression of AMP-activated protein kinase (AMPK), phosphorylated AMPK (p-AMPK), and α-tubulin in (A) Hep3B, (B) C2C12, and (C) 3T3-L1 cells after treatment with 5 or 20 ng/ml macrophage inhibitory cytokine 1 (MIC-1) for 24 h (n = 3). Values are presented as the mean ± SEM. *p<0.05. Three independent experiments were performed. (TIFF) [file pone.0287146.s001.tiff]

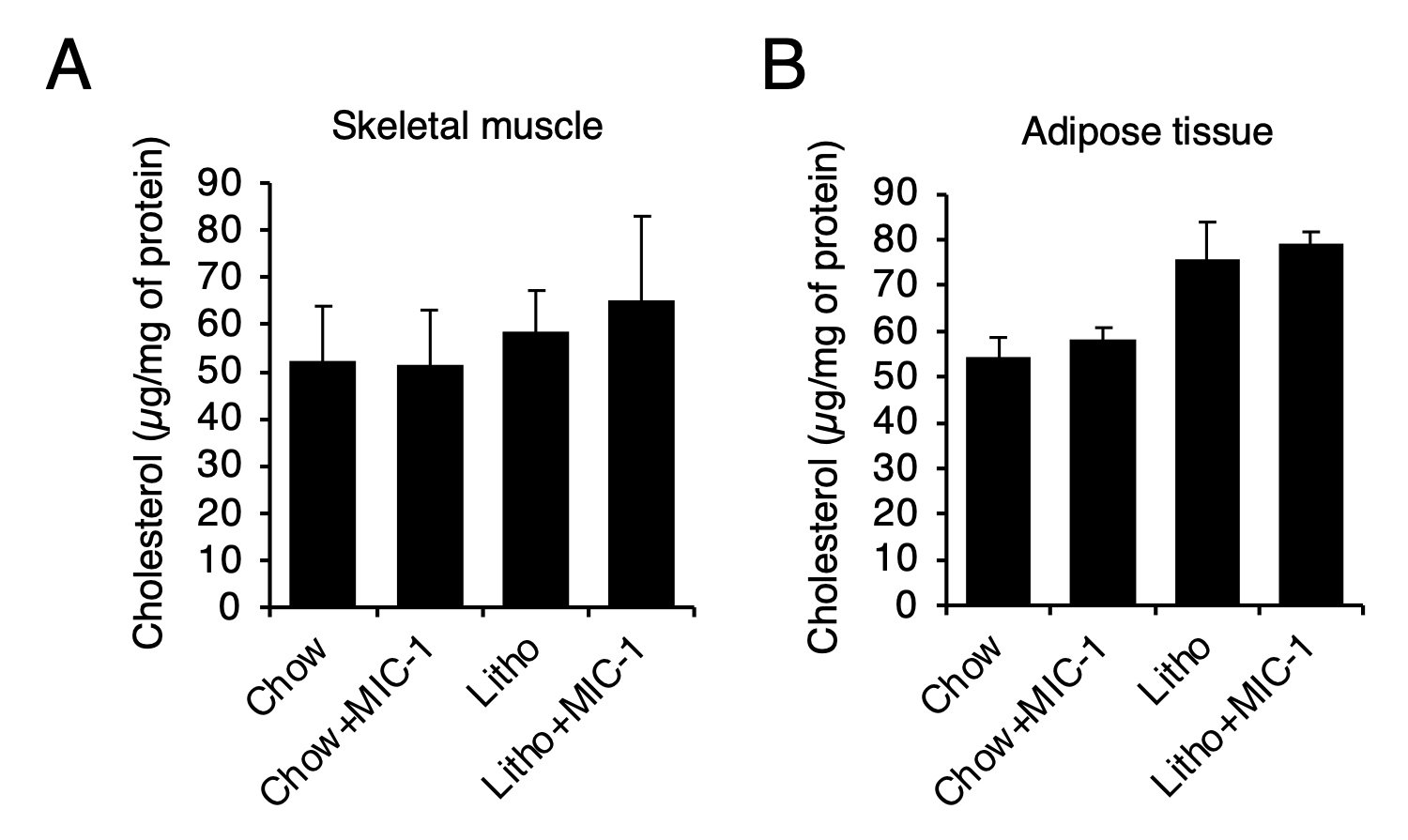

Supplement: S2 Fig — Cholesterol levels were measured in muscle (A) and adipose tissues (B) obtained from mice injected with macrophage inhibitory cytokine 1 (MIC-1; n = 5). Values are presented as the mean ± SEM. Three independent experiments were performed. Chow, standard chow diet; Litho, lithogenic diet. (TIFF) [file pone.0287146.s002.tiff]

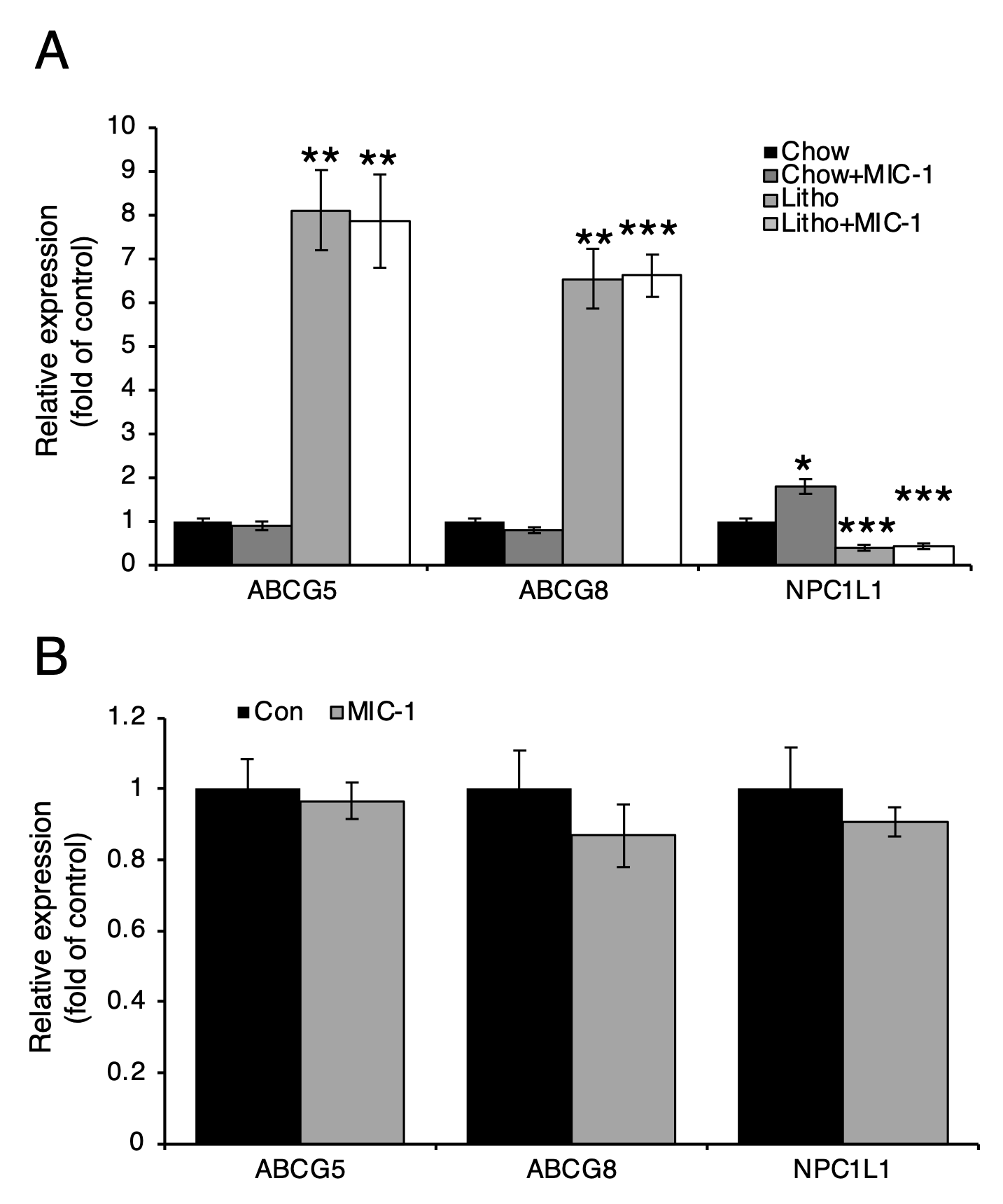

Supplement: S3 Fig — Real-time PCR was used to measure the mRNA levels of ATP-binding cassette superfamily G (ABCG5), ABCG8, and Niemann-Pick C1-like 1 (NPC1L1) in the (A) small intestines of mice injected with macrophage inhibitory cytokine 1 (MIC-1) (n = 5) and (B) Caco2 cells treated with 10 ng/ml MIC-1 for 24 h (n = 5). Values are presented as the mean ± SEM. *p<0.05, **p<0.01, ***p<0.001. Three independent experiments were performed. Chow, standard chow diet; Litho, lithogenic diet; Con, control. (TIFF) [file pone.0287146.s003.tiff]
