# Supplementary material for: Macrophage inhibitory cytokine-1 aggravates diet-induced gallstone formation via increased ABCG5/ABCG8 expression
Source: PLoS One. 2023 Jun 13;18(6):e0287146. doi: 10.1371/journal.pone.0287146 (PMC10263326; doi:10.1371/journal.pone.0287146)
Supplement: S1 Raw images — (PDF) [file pone.0287146.s004.pdf]

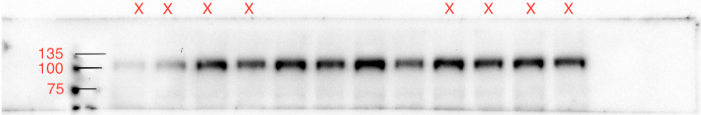
X X X X X X X X
135
100
75

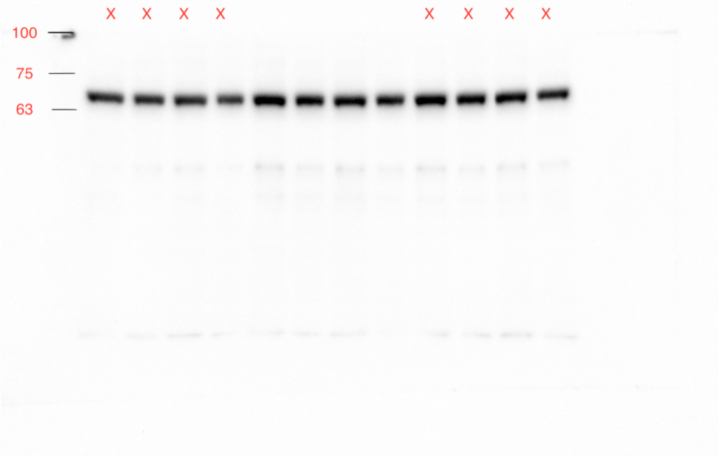
X X X X X X X X
100
75
63

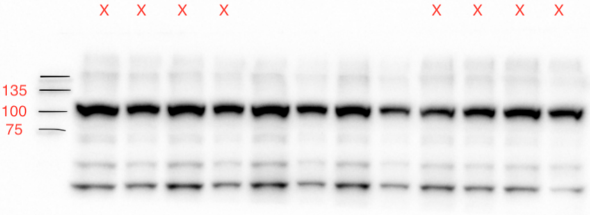
X X X X X X X X
135
100
75

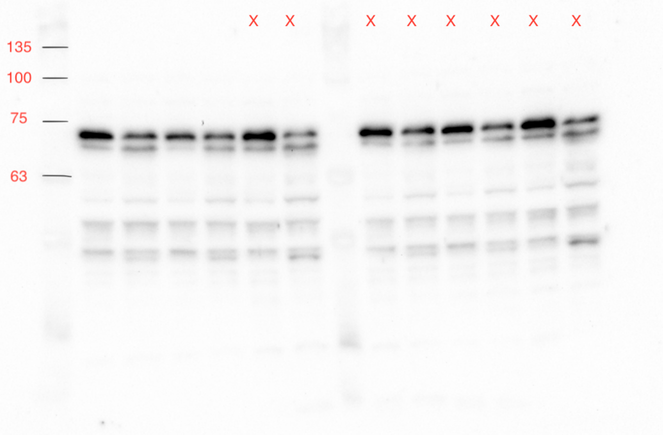

X X X X X X X X
135
100
75
63

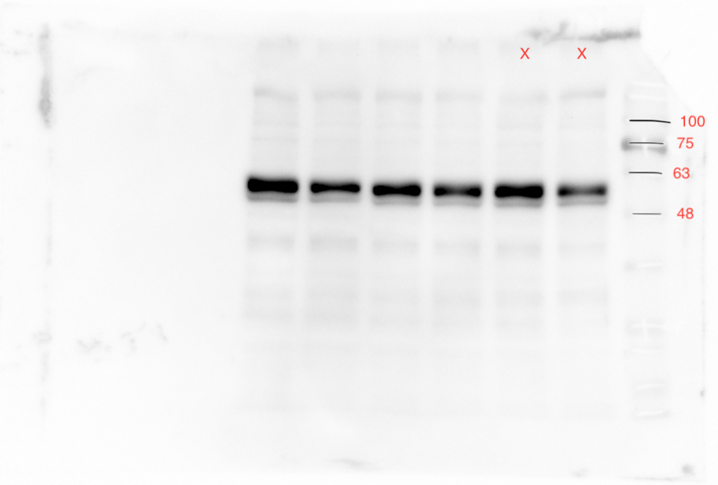
X
X
100
75
63
48

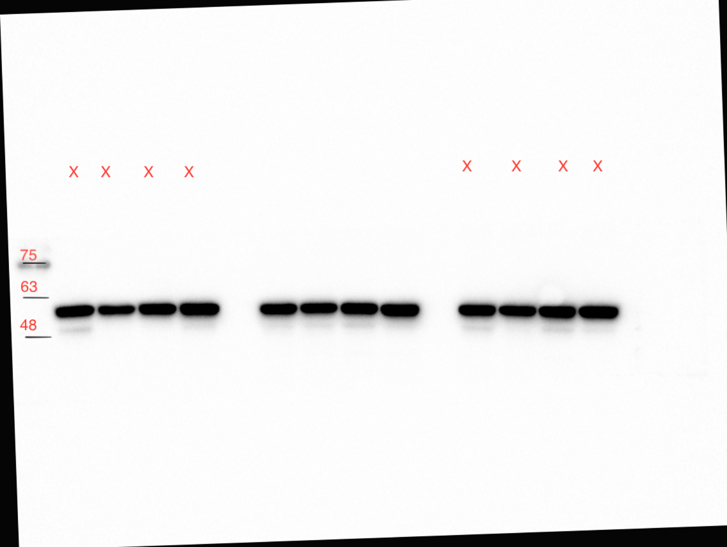
X
X
X
X
X
X
X
X
75
63
48

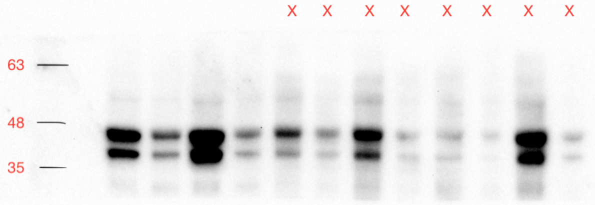

X X X X X X X X
63
48
35

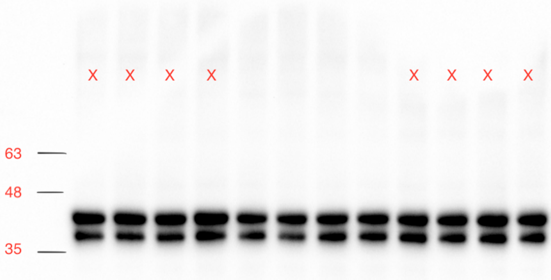
x x x x x x x x
63
48
35

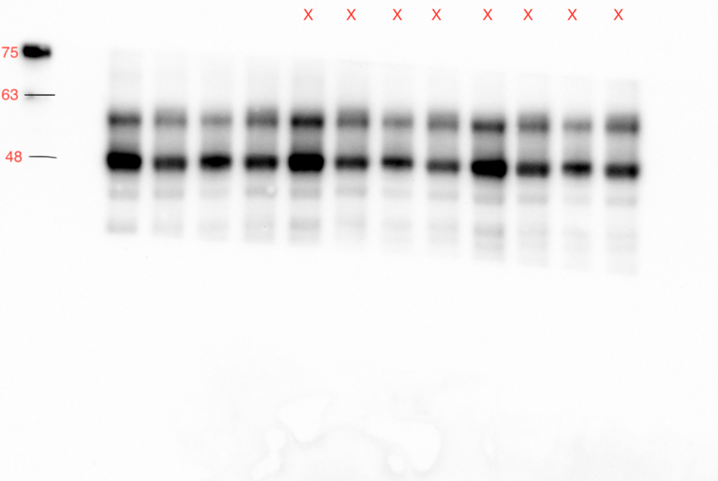

X X X X X X X X
75
63
48

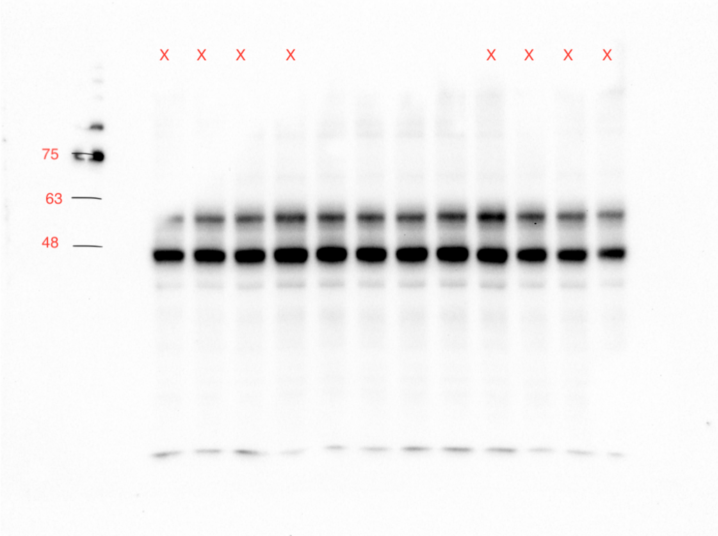

X X X X X X X X
75
63
48

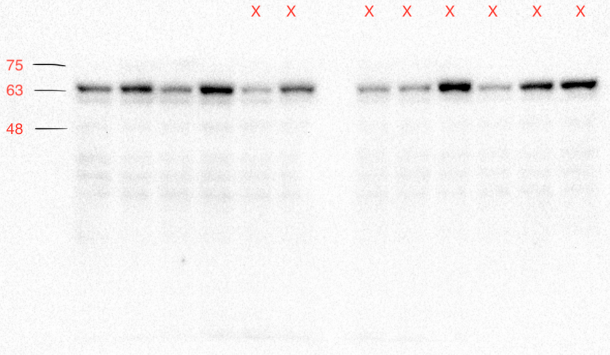
X X X X X X X X
75
63
48

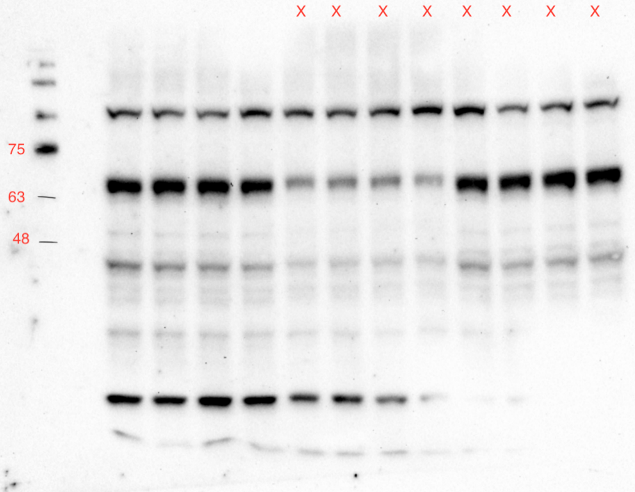
x x x x x x x x
75
63
48

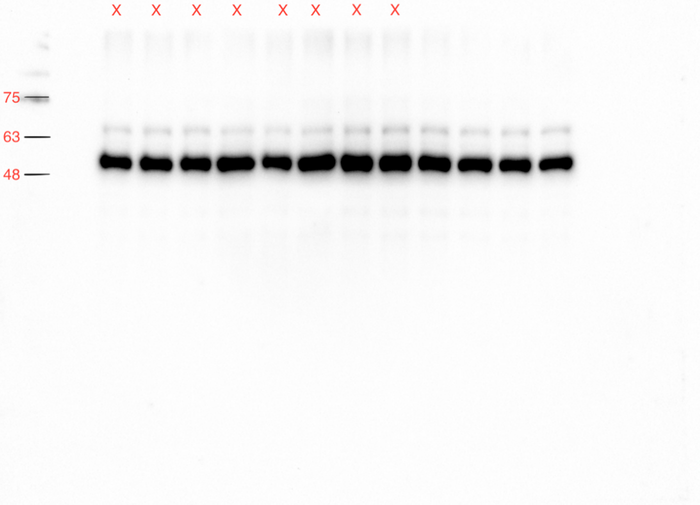
x x x x x x x x
75
63
48

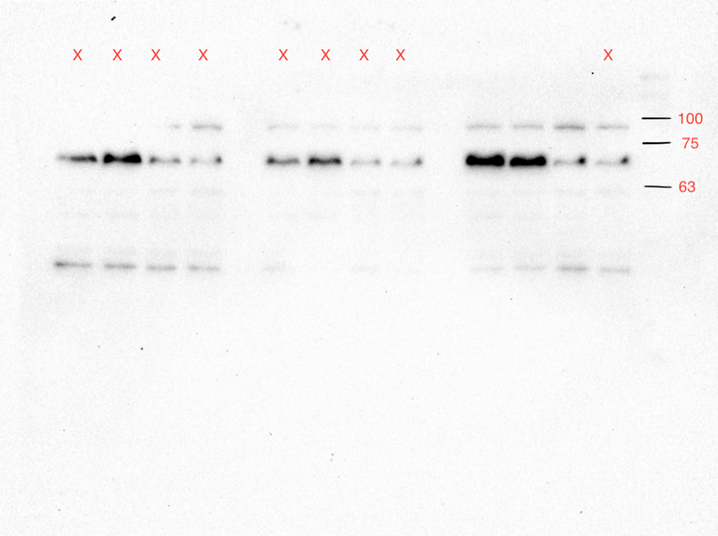

X X X X X X X X X
100
75
63

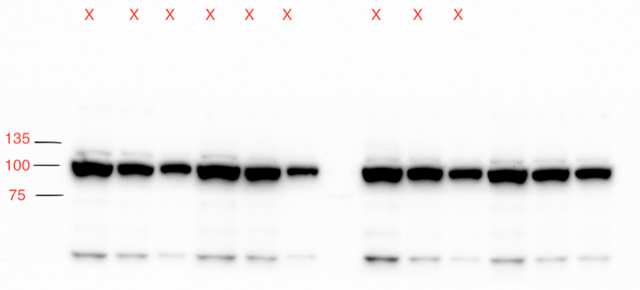
x x x x x x x x x
135
100
75

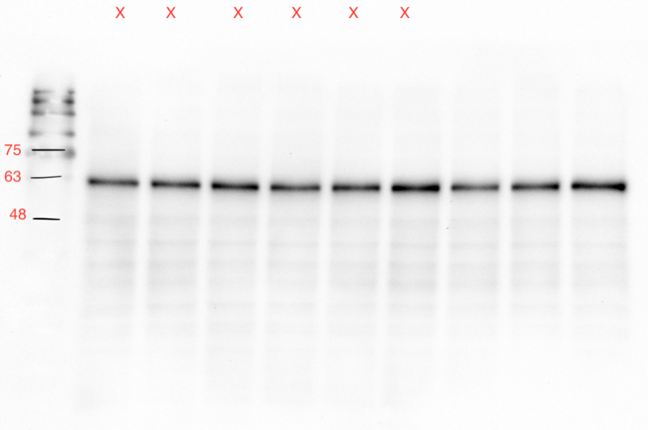
X
X
X
X
X
X
75
63
48

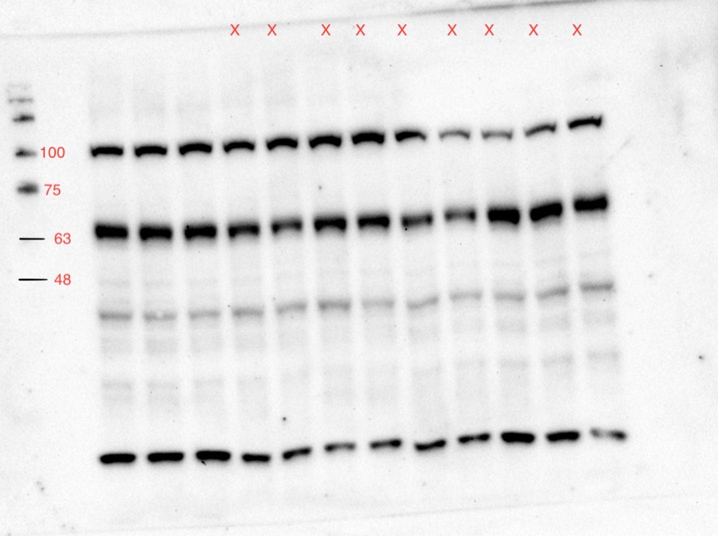
X X X X X X X X X
100
75
63
48

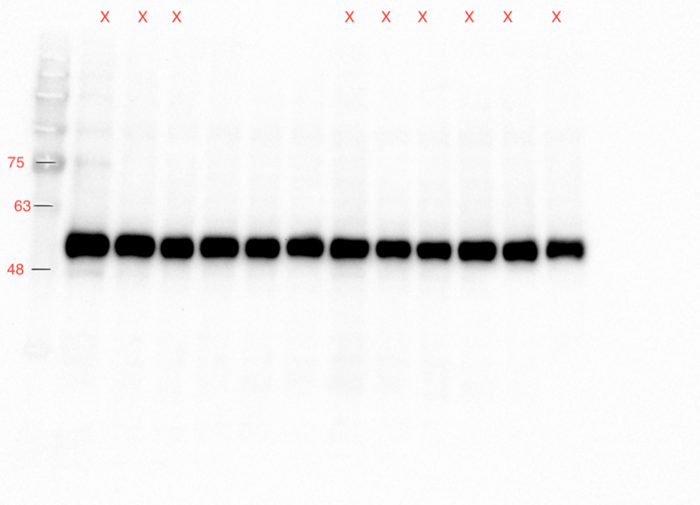
x x x x x x x x x
75
63
48

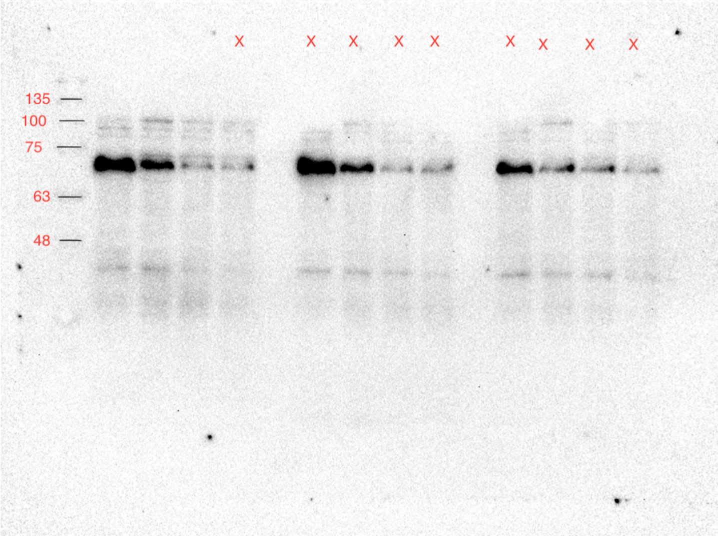
X X X X X X X X X
135
100
75
63
48

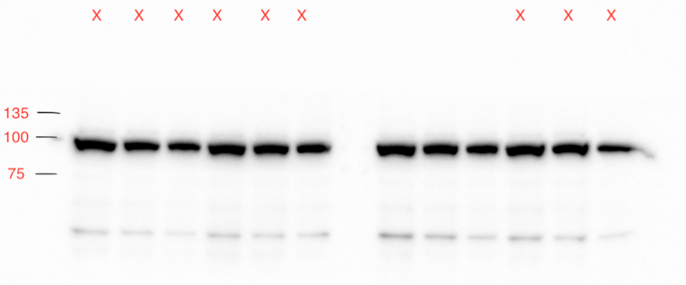

X
X
X
X
X
X
X
X
X
135
100
75

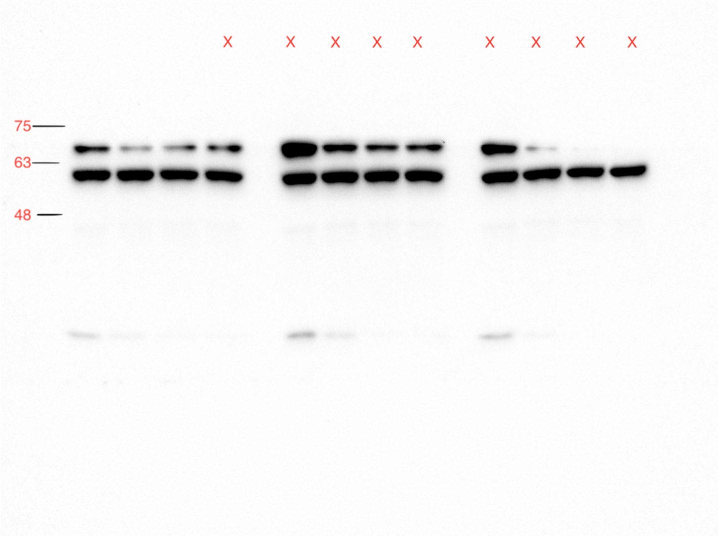
X X X X X X X X X
75
63
48

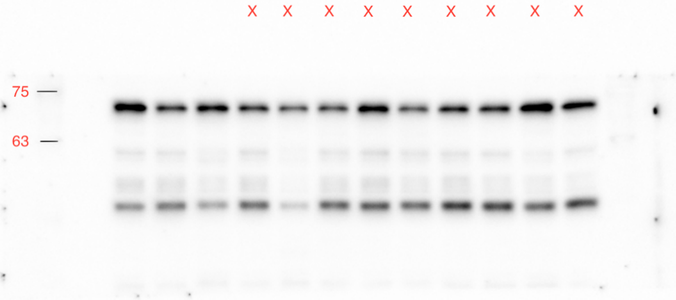
X X X X X X X X X
75
63

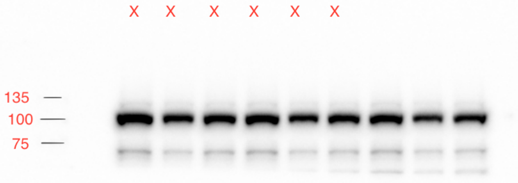
x x x x x x
135
100
75

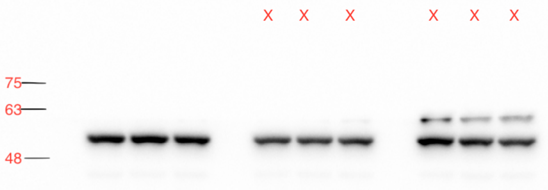

X X X X X X
75
63
48

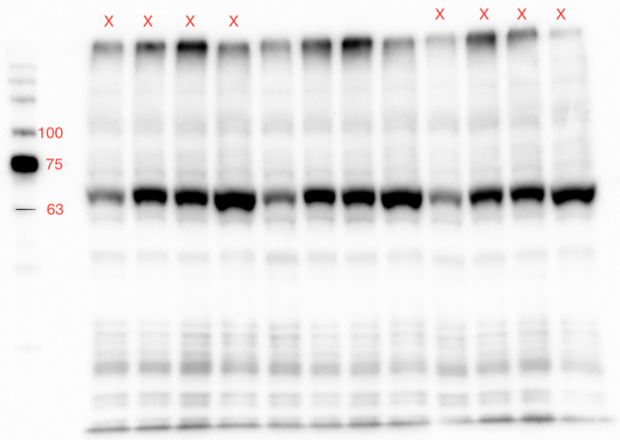

X
X
X
X
X
X
X
X
100
75
63

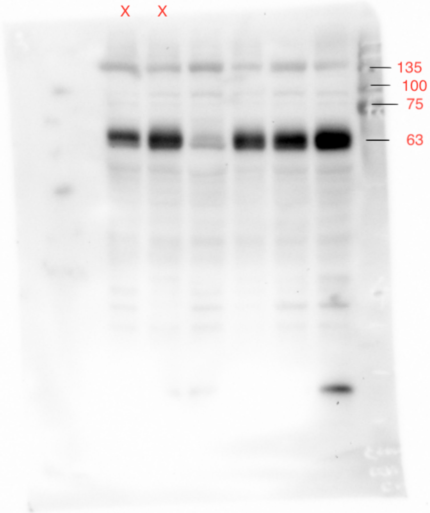
X
X
135
100
75
63

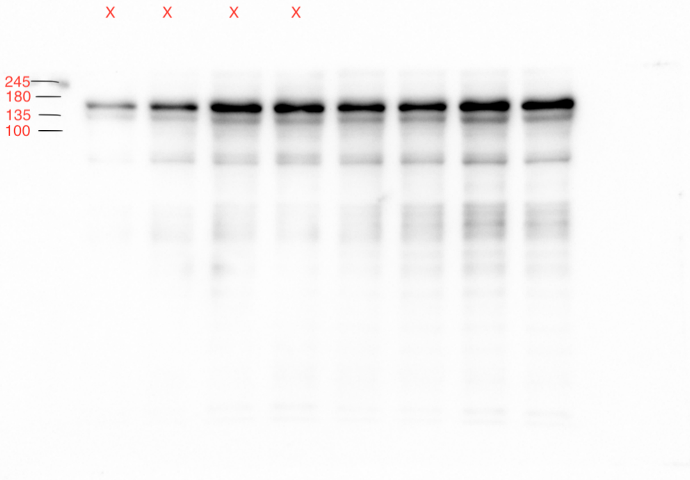
X
X
X
X
245
180
135
100

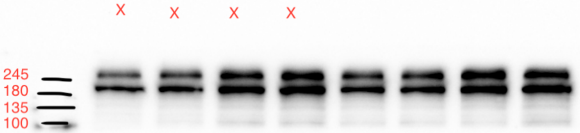
X
X
X
X
245
180
135
100

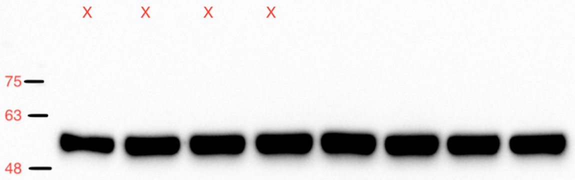

X
X
X
X
75
63
48

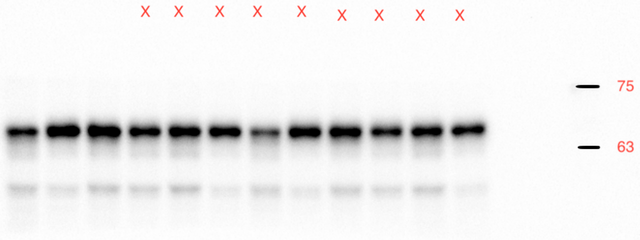
x x x x x x x x x
75
63

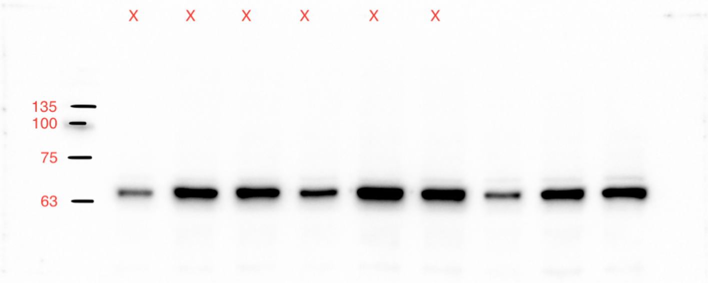
X
X
X
X
X
X
135
100
75
63

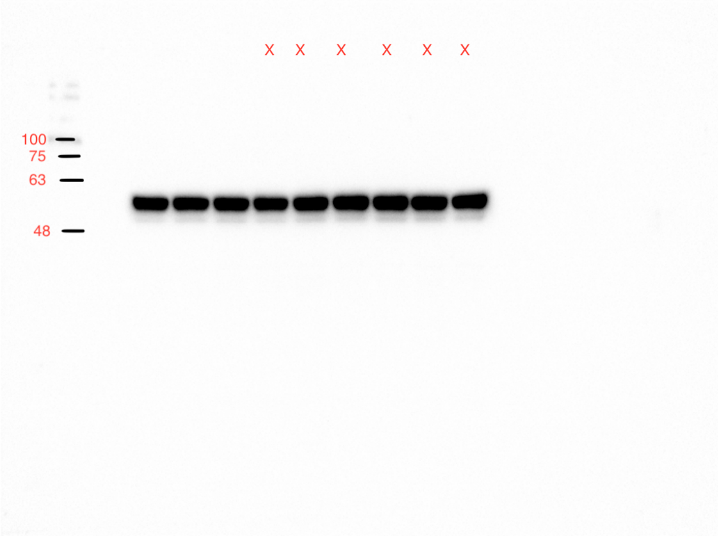
x x x x x x
100
75
63
48

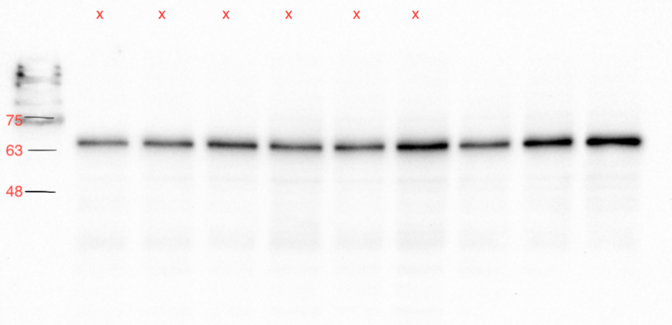
x
x
x
x
x
x
75
63
48

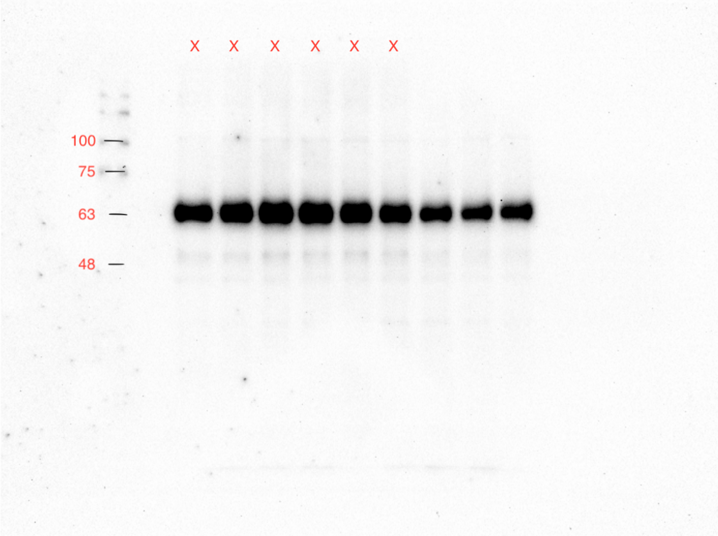

X X X X X X
100
75
63
48

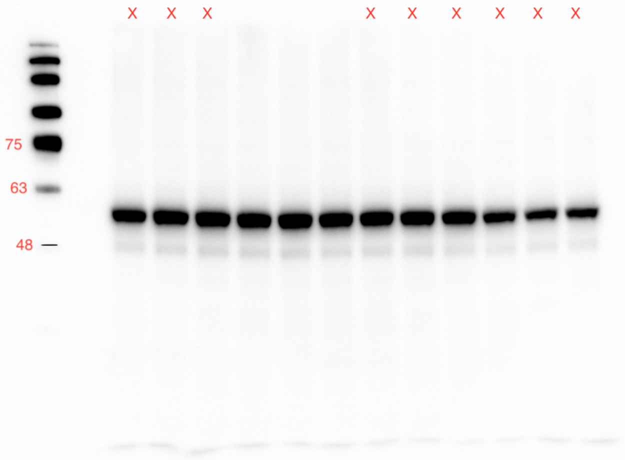
X X X X X X X X X
75
63
48

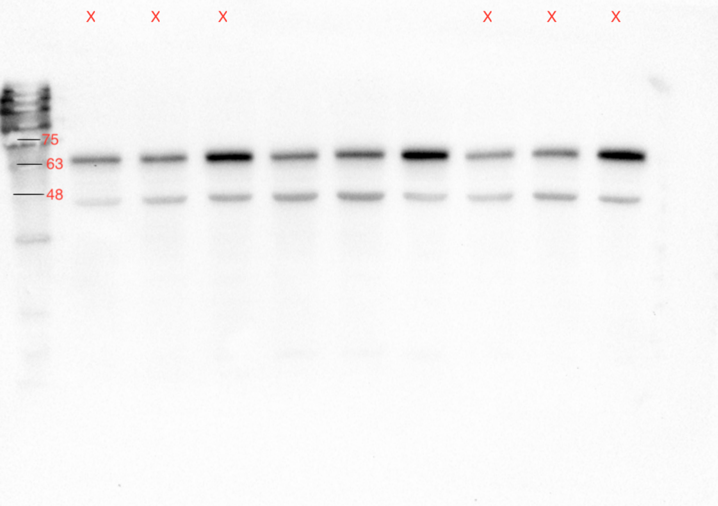
X
X
X
X
X
X
75
63
48

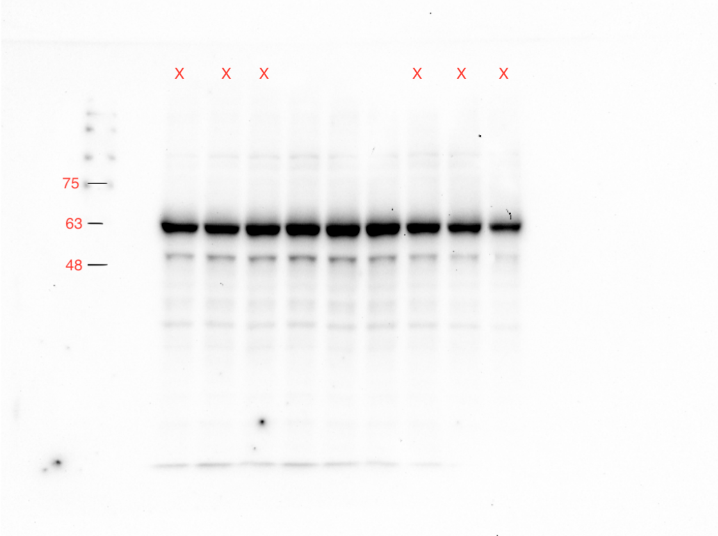
X
X
X
X
X
X
75
63
48

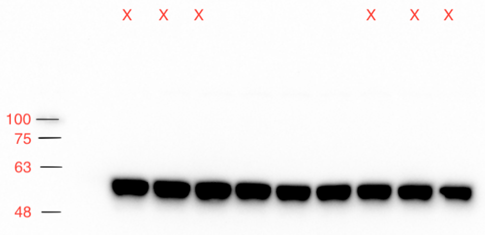
X X X X X X
100
75
63
48

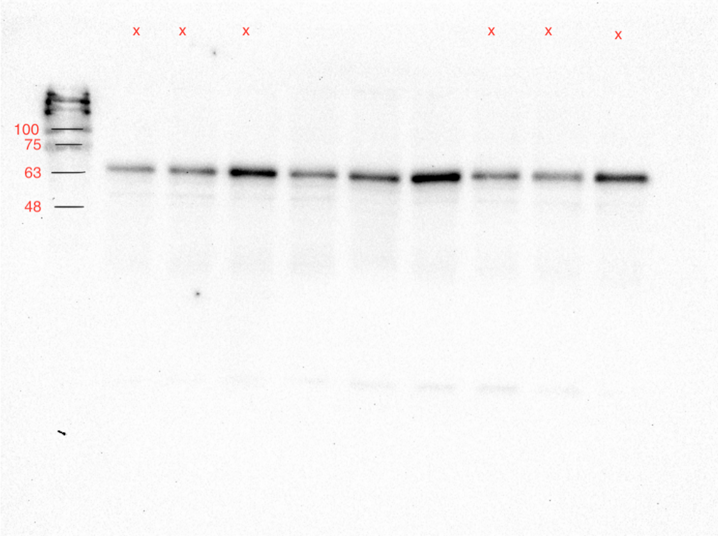

x
x
x
x
x
x
100
75
63
48

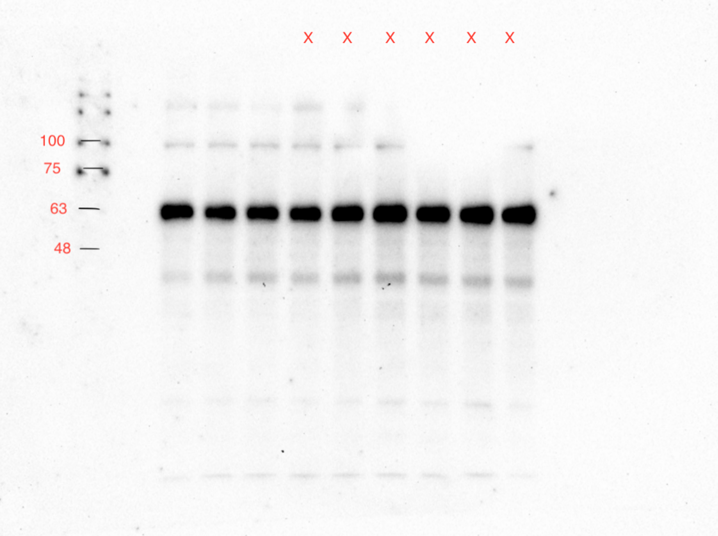
X X X X X X
100
75
63
48

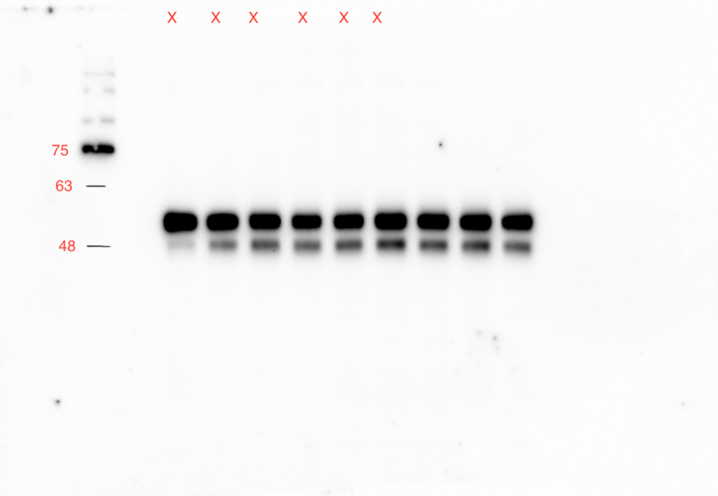
X X X X X X
75
63
48
